# Origin of Public Memory B Cell Clones in Fish After Antiviral Vaccination

**DOI:** 10.3389/fimmu.2018.02115

**Published:** 2018-09-27

**Authors:** Susana Magadan, Luc Jouneau, Maximilian Puelma Touzel, Simon Marillet, Wahiba Chara, Adrien Six, Edwige Quillet, Thierry Mora, Aleksandra M. Walczak, Frédéric Cazals, Oriol Sunyer, Simon Fillatreau, Pierre Boudinot

**Affiliations:** ^1^INRA, Virologie et Immunologie Moléculaires, Université Paris-Saclay, Jouy-en-Josas, France; ^2^Laboratoire de Physique Théorique, CNRS, Sorbonne Université, and École Normale Supérieure (PSL), Paris, France; ^3^Université Côte d'Azur and INRIA, Sophia Antipolis, France; ^4^Sorbonne Université, INSERM, UMR S 959, Immunology-Immunopathology -Immunotherapy (I3), Paris, France; ^5^INRA, Génétique Animale et Biologie Intégrative, Université Paris-Saclay, Jouy-en-Josas, France; ^6^Laboratoire de Physique Statistique, CNRS, UPMC and Ecole Normale Supérieure, PSL, Paris, France; ^7^Department of Pathobiology, School of Veterinary Medicine, University of Pennsylvania, Philadelphia, PA, United States; ^8^INEM, INSERM U1151/CNRS UMR8253, Institut Necker-Enfants Malades, Faculté de Médecine Paris Descartes, Paris, France; ^9^Faculté de Médecine, Université Paris Descartes, Sorbonne Paris Cité, Paris, France; ^10^Assistance Publique – Hôpitaux de Paris (AP-HP), Hôpital Necker Enfants Malades, Paris, France

**Keywords:** antibodies, repertoire, B cells, public response, comparative immunology, fish immunology, RepSeq

## Abstract

Vaccination induces “public” antibody clonotypes common to all individuals of a species, that may mediate universal protection against pathogens. Only few studies tried to trace back the origin of these public B-cell clones. Here we used Illumina sequencing and computational modeling to unveil the mechanisms shaping the structure of the fish memory antibody response against an attenuated Viral Hemorrhagic Septicemia rhabdovirus. After vaccination, a persistent memory response with a public VH5JH5 IgM component was composed of dominant antibodies shared among all individuals. The rearrangement model showed that these public junctions occurred with high probability indicating that they were already favored before vaccination due to the recombination process, as shown in mammals. In addition, these clonotypes were in the naïve repertoire associated with larger similarity classes, composed of junctions differing only at one or two positions by amino acids with comparable properties. The model showed that this property was due to selective processes exerted between the recombination and the naive repertoire. Finally, our results showed that public clonotypes greatly expanded after vaccination displayed several VDJ junctions differing only by one or two amino acids with similar properties, highlighting a convergent response. The fish public memory antibody response to a virus is therefore shaped at three levels: by recombination biases, by selection acting on the formation of the pre-vaccination repertoire, and by convergent selection of functionally similar clonotypes during the response. We also show that naive repertoires of IgM and IgT have different structures and sharing between individuals, due to selection biases. In sum, our comparative approach identifies three conserved features of the antibody repertoire associated with public memory responses. These features were already present in the last common ancestors of fish and mammals, while other characteristics may represent species-specific solutions.

## Introduction

The adaptive immune system provides vertebrates with a unique ability to generate antigen-specific memory cells associated with an increased protection against previously encountered pathogens. Such responses depend on the available immunological repertoire. The term “repertoire” refers here to the V(D)J rearrangements expressed by the lymphocytes of a particular tissue, at a given moment of the life of an individual, and not to the potential diversity of sequences that can be produced from the genomic resources of the organism (1). Immunological repertoires can now be accessed with unprecedented accuracy using high-throughput DNA sequencing (2–4).

The global characterization of the antibody (Ab) repertoires of unchallenged mammals and fish has highlighted the presence of highly frequent clonotypes shared between several individuals (5–9). This observation indicates that repertoires are not simply determined by equally likely random rearrangements of Ig gene segments (2, 10). Thus, certain receptors might be shared between unchallenged controls simply due to their high generation probability.

The sequencing of the IgH repertoires of humans vaccinated against influenza showed that clonotype expansions reflect secreted Ab responses (11, 12). In addition, a dominant set of convergent VDJ rearrangements specific to influenza and shared by the majority of exposed individuals was identified. Such responses made of clonotypes expanded in nearly all individuals are called public responses, and usually contain potent effector clones (13). Public responses are interesting because it is reasonable to assume that they are directed toward the eliciting antigen, while that assumption can generally not be made for individual-specific private responses. The well-studied case of anti-phosphorylcholine public T15 response exemplifies that public responses can play a major role in protection against the targeted pathogen (14, 15). Understanding how public memory clonotypes are selected from the naïve repertoire after an immune challenge might facilitate the development of better universal vaccines. Recently, a few studies have pointed the importance of a genetic pre-determination of the rearrangement probability as a mechanism affecting the establishment of public memory responses after an immune challenge (5, 16, 17).

Public Ab responses are observed in evolutionarily distant species since they are also found in fish, which evolved in parallel to tetrapods over the past 400 million years. We previously identified a public IgM effector response to an attenuated strain of the rhabdovirus Viral Hemorrhagic Septicemia Virus (VHSV) in isogenic rainbow trout (18). In fish, IgM^+^ B cells are complemented by IgT^+^ B cells (19), which constitute a distinct lineage since these two Ig are produced from alternative rearrangements, and B cells do not undergo isotype switch recombination. IgM acts primarily as a systemic Ig and is the major Ig class in the serum, while IgT is mainly — but not exclusively—specialized in mucosal immunity (20) and in the control of the gut microbiota (21). In spleen, 70–80% of B cells express IgM, and 20–30% IgT. IgM^+^ B cells are the main source of circulating virus-neutralizing Abs following VHSV infection (18). However, a response was also detected for IgT after a prime/boost with VHSV, which did not show evidence of a public component (18).

In this work, starting with the characterization of the pre-vaccination repertoire, we found that IgM repertoires of naïve fish included highly abundant fish-specific clonotypes, as well as less abundant clonotypes with a higher degree of sharing between fish. By contrast, IgT repertoires were richer in shared clonotypes, and the most abundant ones were much less frequent than for IgM. Following vaccination, fish established persistent public IgM clonotype expansions in spleen for several months after the clearance of the virus. We sought to elucidate the properties of these clonotypes, to gain some insight into the selection processes associated with the establishment of public memory cells. We demonstrate that both the statistics of Ig gene rearrangement and selection processes prior to vaccination contribute to the generation of repertoires in unvaccinated fish that yield the public memory responses.

## Materials and methods

### Fish vaccination and ethical statement

Rainbow trout were raised, vaccinated against VHSV and boosted in the fish facilities of INRA (Jouy en Josas, France). This study was carried out in accordance with the recommendations of the European Union guidelines for the handling of laboratory animals (http://ec.europa.eu/environment/chemicals/lab_animals/index_en.htm). All animal work at INRA was approved by the Direction of the Veterinary Services of Versailles (authorization 78-28) as well as fish facilities (authorization B78-720), and the experimental protocols were approved by the INRA institutional ethical committee “Comethea” (permit license number #15-60).

### Elispot and virus neutralization assay

VHSV specific IgM secreting cells were determined in head kidney using *ex vivo* ELISPOT (22) and Supplementary Methods. Virus neutralization assay with complement addition was performed in 12-well plates as previously described (18). The neutralizing titer was calculated as highest trout serum dilution causing a 50% reduction of the average number of plaques in control cultures inoculated with control trout serum, complement and virus.

### CDR3 length spectratyping analysis and preparation of illumina MiSeq libraries

CDR3 length spectratyping analysis was performed as described in Castro et al. (18), and CDR3 length profiles were generated by GeneMapper (Applied). Libraries for Illumina deep sequencing were prepared as described in Vollmers et al. (11). For cDNA barcoding, the primers used for second strand cDNA contained 15 random nt (Figure S1 and Supplementary Methods).

### Sequencing and data analysis

Sequencing consisted in paired-end 2 × 300 pb runs, using a MiSeq instrument (Illumina) and the MiSeq Reagent Kit v3 (600 cycles) (Illumina). Sequencing analysis and annotation, estimation of error rate, and normalization by subsampling, as well as validation of our barcoded IgH cDNA sequencing approach, are described in Supplementary Methods.

Sequence data were registered in the BioProject ncbi database with the SRA accession number: SRP128087.

### Computational model of IgH VDJ rearrangements

We employed an existing computational tool suite, IGoR (23), to construct a generative probabilistic model of the IgH VDJ recombination process and generate corresponding synthetic receptor sequences. The parameters were inferred for each VHC combination sequenced in this work, from the corresponding sets of non-productive sequences. IGoR can then be used to generate synthetic nucleotide sequences, which can readily be translated into amino-acid sequences and compared between synthetic datasets (i.e., generated by the model). A detailed description of the model is provided in Supplementary Methods.

## Results

### Vaccination with attenuated VHSV induces persistent public IgM response in spleen

**Figure 1:**
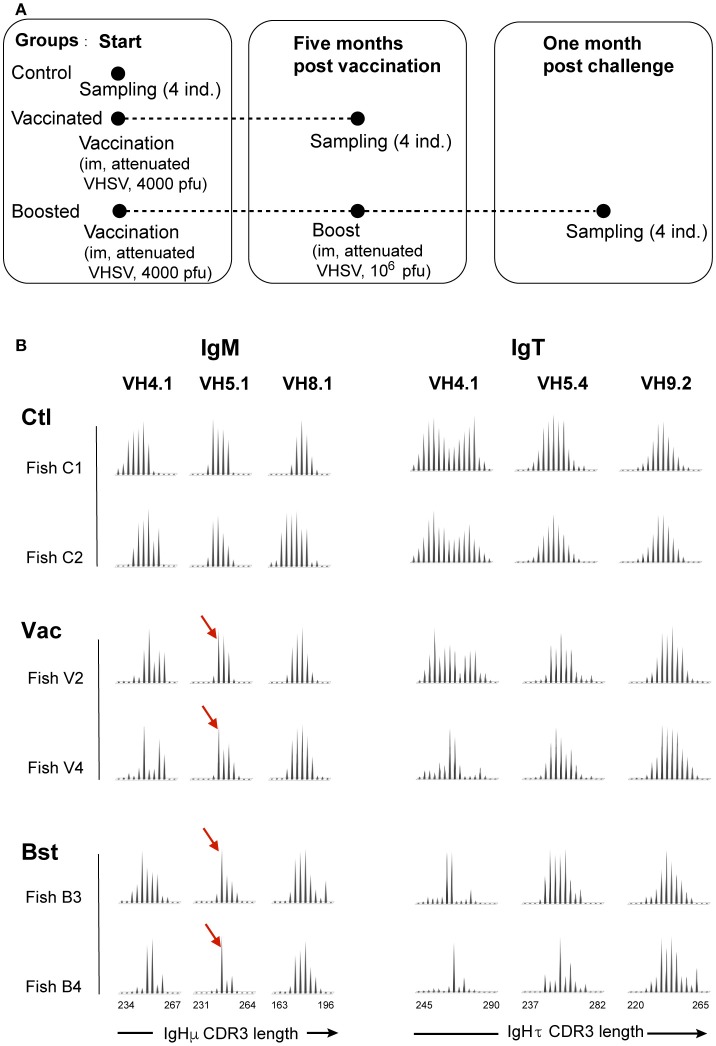
Spectratyping analysis identifies persistent Ab memory responses to VHSV. **(A)** Time line of sampling, vaccination and boost experiments. Groups of six fish were constituted. **(B)** Typical CDR3 length profiles of μ and τ transcripts for selected VH in the spleen of controls (“Ctl”), vaccinated fish analyzed 5 months post-injection (“Vac”), and boosted fish analyzed month later (“Bst”). Profiles for other VH are shown in Figure S3. The red arrows denote the main peak of the putative public response. VH primers used are indicated at the top of the figure. Y-axis, fluorescence arbitrary units; X-axis, length of the run-off products; same scale for IgM and IgT.

Long-lasting humoral immune responses can be induced in fish after immunization (22, 24). Five months after vaccination with an attenuated VHSH strain (Figure 1A), rainbow trout had elevated serum neutralizing Ab titers (Table S1A), long after the elimination of the virus, which was not detectable by qPCR already 1 month post-injection. We analyzed the spleen B cell repertoire at 5 months post-vaccination to characterize the long-term persisting reactive B cells, which we called “memory” following A Radbruch's definition in Farber et al. (25). This was first performed with a global cost-effective CDR3 spectratyping of all expressed combinations of heavy chain variable (VH) and constant (C) genes, to identify the Ig gene segments implicated in the response and therefore relevant for further analysis by high-throughput sequencing (Supplementary Methods). In fish all VH segments can recombine with either μ or τ DJC units, while IgM and IgT have distinct repertoires of DH and JH genes (Figure S1). An isogenic trout clone was used to avoid genetic background heterogeneity (18).

The IgM repertoires of vaccinated fish had altered profiles for the VH1, VH4, and VH5 families (Figure 1B, Figure S3). The altered profile of VH5 was the only one found in all vaccinated individuals (Figure 1B), suggesting that it may contain a persisting public component, while the responses associated with VH1 and VH4 varied from fish to fish. Notably, the VH5 family was previously found to be implicated in the VHSV-specific public effector B cell response (18). The IgT repertoire was also modified 5 months after vaccination, with most individuals displaying skewed VH4Cτ profiles compared to unvaccinated controls, even though no common peak was identified (Figure 1B, Figure S3).

The implication of only a few VH families in the long-term B cell response is in contrast to the previous identification of broad Ig repertoire alterations affecting almost all VH families shortly after two infections at a 3-weeks interval with the same rhabdovirus (18). This suggests that the spectratype analysis performed 5 months after vaccination may have identified only a part of the memory compartment. To look for other rearrangements that were potentially implicated in the memory response, vaccinated fish were boosted at 5 months post-vaccination, and their repertoire was analyzed 1 month later (Figure 1A). The boost induced a >10-fold increase of the frequency of anti-VHSV IgM-secreting cells in pronephros, proving that a secondary response occurred (Table S1B), even though it did not lead to a significant increase of neutralizing Ab titers (Table S1A). Importantly, the response observed in the spleen after the boost remained restricted to the VH families that were already identified 5 months post-vaccination (Figure 1B, Figure S3).

We conclude from these results that the vaccination induced a long-lasting IgM memory response associated with only a few VH families, which persisted beyond the elimination of the virus.

### Distinct properties of the most abundant IgM and IgT clonotypes in unvaccinated controls

Having validated that it was possible to identify a memory B cell response in fish, potentially comprising a public VH5Cμ response, we then examined the clonal structure of the VH5Cμ response using Illumina deep sequencing. We also investigated the private VH4Cμ and VH4Cτ responses, as well as some VHC combinations that did not obviously contribute to the response (VH8Cμ, VH5Cτ, and VH9Cτ) as controls. Sequencing was performed in unvaccinated control (Ctl), vaccinated (Vac), and boosted (Bst) fish with 4 individuals per group. We used a consensus read sequencing approach based on the incorporation of a unique random barcode serving as molecular identifier in each cDNA molecule, as described for human IgH (11) [see Figures S1, S2 and Supplementary Methods about molecular identifiers (MID)]. We define a clonotype as a specific combination of C (μ or τ), VH, JH, and CDR3 amino acid sequence. We first describe results obtained for unvaccinated controls, which provide a benchmark for the analysis of the changes induced by the vaccination and the boost.

Individual fish datasets obtained for each VHC combination were normalized by random subsampling of 7,000 clonotypes without replacement to quantitatively compare all fish together despite varying sequencing depths, while preserving the clonotypes' relative frequencies. Ten subsamplings were performed, and the average results were analyzed (see Supporting methods for details). A comparison of the number of distinct IgM and IgT clonotypes in unvaccinated controls suggested that the repertoires were more diverse for IgT than for IgM for all VH (Table S2A). We counted the number of IgM and IgT clonotypes found in at least 3 individuals, which were labeled as “highly shared” (HS). The numbers of HS clonotypes were higher for VHCτ than for VHCμ sequences, except for VH5Cμ (Table S2B). These two observations suggested that the IgT repertoires were richer in shared clonotypes of small abundances, while IgM repertoires included clonotypes of large abundances that were fish-specific. This last statement was further supported by the abundance distribution of clonotypes present in 1, 2, 3 or all unvaccinated control fish (Figure S4A). It is nonetheless noteworthy that the VH5Cμ repertoire contained a higher number of HS clonotypes of small abundance compared to the other IgM repertoires.

**Figure 2:**
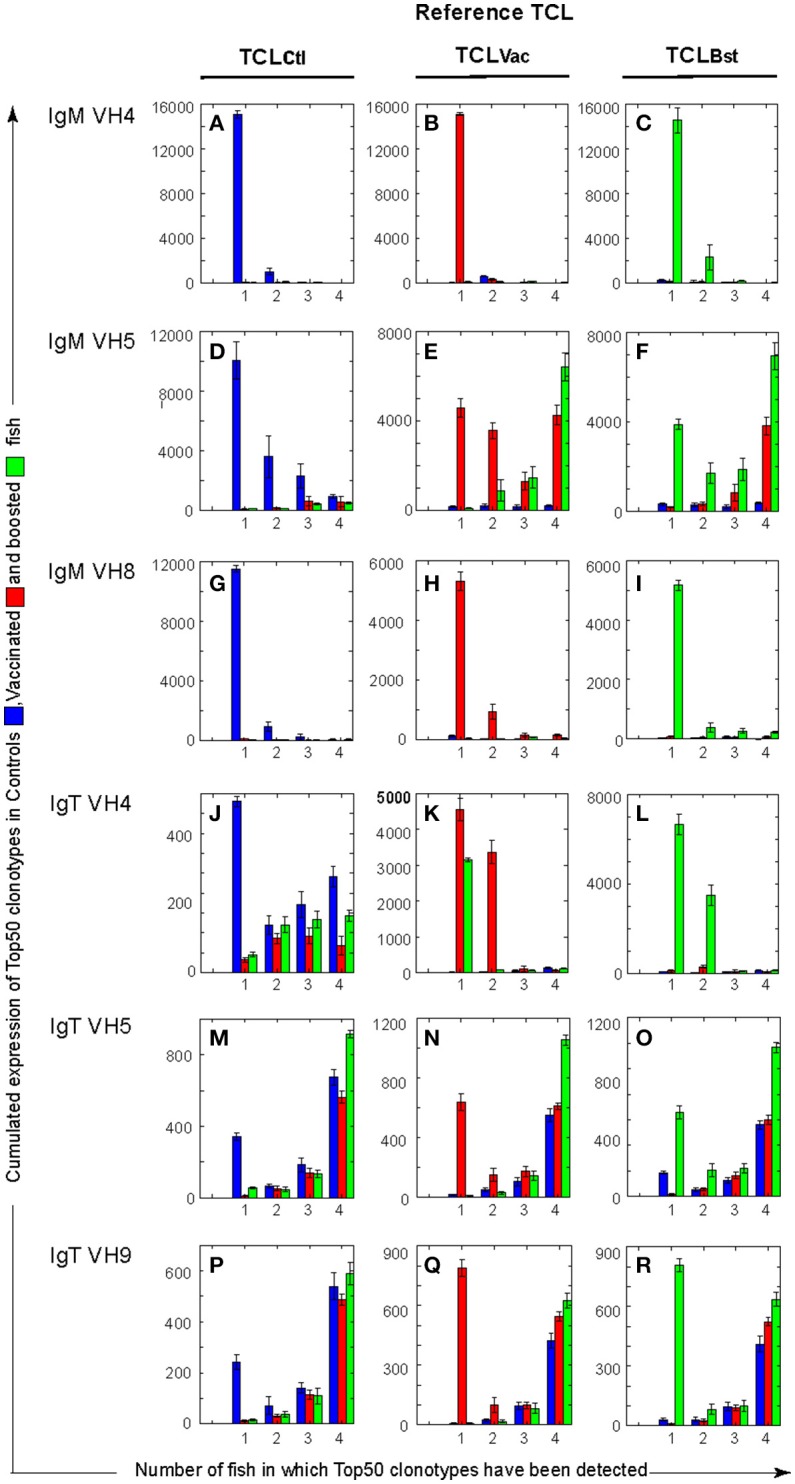
Cumulative expression of Top50 clonotypes shared by *n* individuals within each group (Ctl, control; Vac, vaccinated; and Bst, Boosted) unveils differences between VH and isotypes. For each TCL (TCLCtl, (**A,D,G,J,M,P**); TCLVac, (**B,E,H,K,N,Q**); TCLBst, (**C,F,I,L,O,R**)), bar plots showing the cumulative expression of TCL clonotypes found in individual subsample(s) from *n* fish (*n* = 1, 2, 3, 4). For example, in **(A)** blue bars show the cumulative counts of TCLCtl clonotypes in fish from the control group, while red (respectively green) bars show TCLCtl cumulative expression in fish from the vaccinated (respectively boosted) group. Similarly, in **(B)** red bars show the cumulative counts of TCLVac clonotypes in vaccinated fish, while blue (respectively green) bars show TCLVac cumulative expression in control and boosted groups, respectively. Bars are computed from the average values corresponding to top clonotypes found in 1–4 fish, over 10 subsamplings of 7,000). The standard deviations are shown as error bars.

We next focused on the most abundant clonotypes for each VHC combination in each fish. To this end, we considered the 50 or 100 most abundant clonotypes (called “Top50” and “Top100” clonotypes thereafter). For each group (Ctl, Vac, and Bst), the 4 sets of “Top50” clonotypes from each of the 4 fish in the group were aggregated to obtain lists of non-redundant clonotypes called Top Clonotype Lists (TCL)_Ctl_, TCL_Vac_, or TCL_Bst_. The total number of clonotypes in a TCL was thus between 50 (if all were shared by the 4 fish of the group), and 200 (if none were shared) for the Top50 case. We then analyzed for each clonotype of a TCL in how many fish of a group (Ctl, Vac, or Bst) it was found. The distributions of TCL_Ctl_ clonotypes are represented in the left column in Figure 2, Figure S5A. Individual fish datasets, for each VHC combination, were normalized as described above. The clonotype sharing was also studied for the Top100 (Figure S5B), and for the whole set of subsampled sequences (Figure S5C).

In the unvaccinated control group, most of the VH4Cμ and VH8Cμ TCL_Ctl_ clonotypes were found in only one fish (Figure S5A,a,g, blue bars). These clonotypes were not found in any other fish of the vaccinated or boosted groups, indicating that they were private expansions found in a single fish. In contrast, 53 out of 190 VH5Cμ TCL_Ctl_ clonotypes were shared by two or more fish (Figure S5A,d, blue bars). The total number of clonotypes in the TCL_Ctl_ was lower for IgT rearrangements than for IgM (197 for VH4Cμ, 189 for VH5Cμ, 198 for VH8Cμ, vs. 146 for VH4Cτ, 110 for VH5Cτ, and 103 for VH9Cτ), indicating a higher degree of sharing of the most abundant IgT clonotypes between fish for IgT compared to IgM. A substantial fraction of the IgT TCL_Ctl_ were found in several fish, i.e., 70% for VH5Cτ, 66% for VH9Cτ, and 45% for VH4Cτ (Figure S5A,j,m,p, blue bars). As expected from this elevated degree of sharing, a significant fraction of the IgT TCL_Ctl_ were also found in the vaccinated (48% for VH4Cτ, 68% for VH5Cτ, and 68% for VH9Cτ), and boosted (56% for VH4Cτ, 75% for VH5Cτ, and 72% for VH9Cτ) fish (Figure S5A,j,m,p, red and green bars).

Top100 clonotypes were shared in a very similar manner (Figure S5B) as Top50 clonotypes, with close to double the number of shared clonotypes compared to the Top50 lists. In contrast, when all 7,000 subsampled clonotypes were taken into account (Figure S5C), only a small fraction were shared by two or more individuals, even for the VHC combinations that share a significant numbers of Top clonotypes, i.e., VH5Cμ (6%), VH4Cτ (2%), VH5Cτ (5%), and VH9Cτ (4%) This shared fraction therefore mostly involved the most abundant clonotypes (i.e., included in the Top 50 lists), in unvaccinated fish.

To assess the overall contribution of the Top50 clonotypes to the repertoire in unvaccinated controls, we analyzed the cumulative expression of these TCL_Ctl_ within each group. The cumulative counts of the Top50 clonotypes for the 4 naïve control fish were on average >10,000 for all VHCμ combinations, out of a total number of 28,000 sequences analyzed (4 fish × 7,000 MID subsampled per fish). Highly frequent clonotypes thus represent a large fraction of the IgM transcripts in the control group. In contrast, Top50 clonotypes did not represent a large part of the IgT repertoires, with averaged cumulative counts of about 1,000 (Figure 2A, left column, blue bars). Distributions of cumulative expression of Top100 clonotypes showed only a small increase compared to Top50 clonotypes (Figure S6A), indicating that Top50 clonotypes account for most of the cumulative counts (see also Figure S6B for all clonotypes).

In summary, in unvaccinated control fish, the most frequent clonotypes represent a considerable fraction of IgM transcripts and differ from fish to fish (except for VH5), while the most frequent IgT clonotypes account for only a small fraction of the expressed repertoire and are generally shared between individuals (Figure S4B). Our results thus reveal different degrees of sharing among IgM and IgT repertoires in the spleen of naïve unvaccinated control fish, and point to the peculiarity of the VH5Cμ repertoire.

### Sharing of IgM and IgT top50 clonotypes in unvaccinated controls is determined by selection rather than at the rearrangement level

These differences between IgM and IgT Top50 clonotypes, i.e., their contrasted degree of sharing between unvaccinated controls, may already exist in the clonotypes that emerge from the recombination process, before any selection. To assess this possibility, we constructed a model of the recombination machinery using the IGoR software (23). IGoR can be used to generate “synthetic” nucleotide sequences, which can readily be translated into amino-acid sequences and used for comparisons. It is based on the statistics of IgH VDJ rearrangements computed using the non-productive (either out-of-frame or with an in-frame premature stop codon) junctions of our dataset (see Supporting methods), which were not subjected to selection since they were not expressed at the protein level. The model was parametrized by the probabilities of the events that form a recombination event: the choice of VH, DH, and JH segments as well as the insertion and deletion profiles at the VD and DJ joints. DH and JH gene usage varied across VH family and Ig isotype, while insertion and deletion profiles were largely similar, with the exception of VH5Cμ for which insertions at the VD junction were smaller (Figures S7A,B).

To evaluate the degree to which the difference in IgM and IgT repertoire diversity was determined by the rearrangement process, we estimated IgM and IgT diversity from the distribution of clonotype generation probabilities (*P*_gen_) obtained from the repertoires generated by IGoR. We quantified diversity using the entropy, *S*, a well-established diversity measure expressed in units of bits (26). The entropy estimates (27) varied by < 15% across the various VHC combinations (Table S2C). This is consistent with the observation of similar deletion and insertion profiles at the VD and DJ joints across VHC combinations (Figure S7B), as previous work (10, 28) has shown that the diversity does not arise mainly from the variety of VDJ combinations but from the deletions and insertions.

Thus, the model predicts similar diversity for IgM and IgT. We infer from this result that the observed difference in diversity between IgT and IgM naive repertoires (Table S2A) is not pre-determined by rearrangements, but rather results from B cell selection.

**Figure 3:**
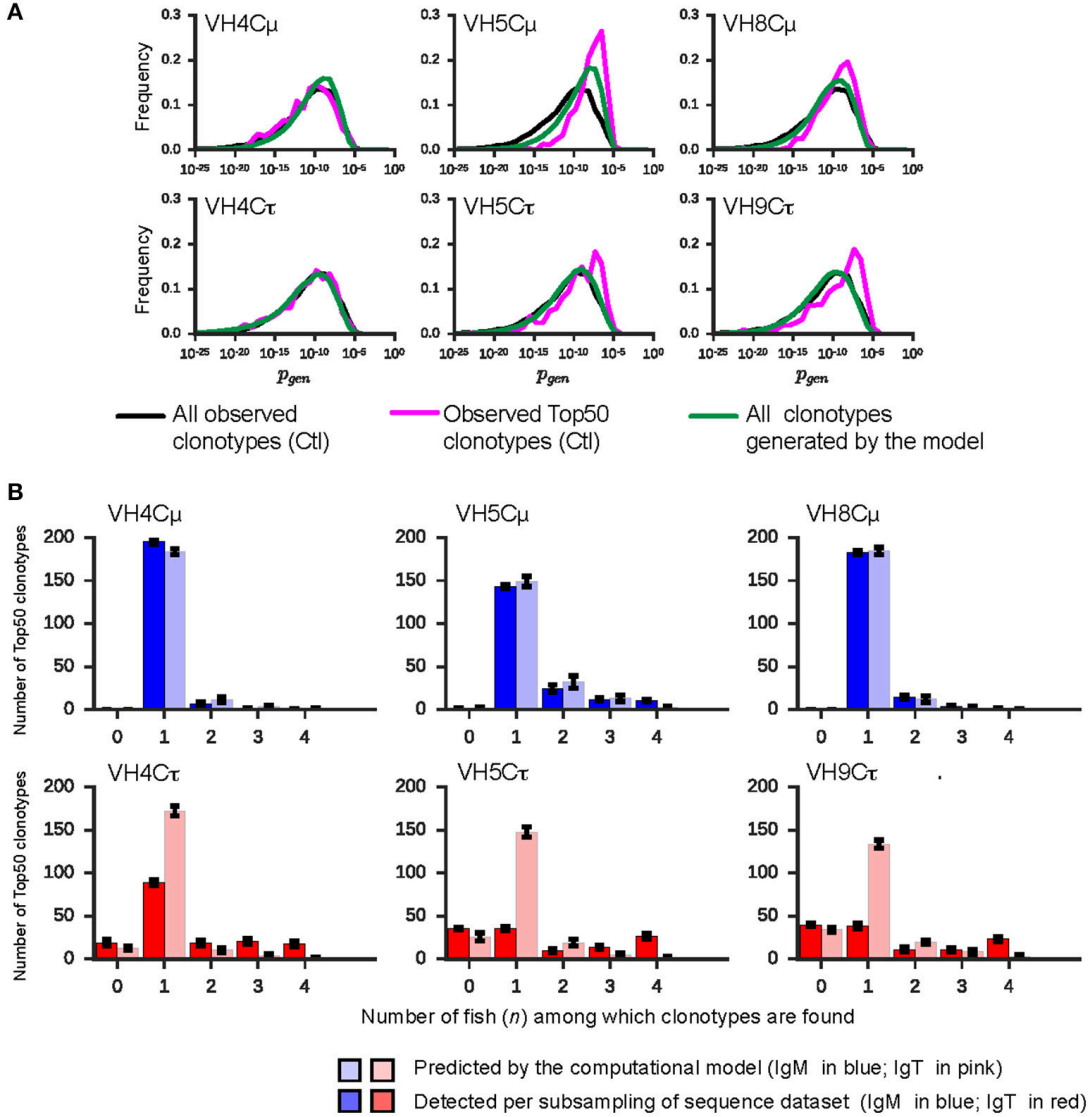
The sharing of Top50 clonotypes between individuals is determined by selection. **(A)** Distribution of Pgen for all clonotypes from controls (black line), for Top50 clonotypes of controls (i.e., TCLCtl; pink line), and for all sequences generated by the computational model (green line). **(B)** Histograms of the sharing of TCLCtl clonotypes. Left (right) bars show the numbers of measured (respectively predicted) TCLCtl clonotypes shared among n individual measured (predicted) repertoires (*n* = 0, 1, 2, 3, 4) out of 4. IgM is colored in blue; IgT is colored in red. Error bars for the rearrangement model correspond to standard error values across sampled repertoires. Error bars for the sequencing data correspond to the standard deviations of 10 subsamplings of 7,000.

We next used this model to address whether the different sharing patterns of TCL between IgM and IgT may be explained, at least partly, by the different probability of generation (*P*_gen_) of the frequent clonotypes across VHC combinations. The model was therefore used to evaluate the *P*_gen_ of observed TCL_Ctl_ junctions. Figure 3A shows that for four VHC combinations, the Top50 clonotypes from controls (purple line) are more likely to be generated via the recombination process, compared to the *P*_gen_ distributions of all observed clonotypes from naive controls (black line), which are fairly similar across VHC combinations (Figure S7C). These *P*_gen_ distributions are in agreement with the model prediction for all possible junctions (green line in Figure 3A). It is however difficult to conclude that the rearrangement process favors Top50 clonotypes in general because such a bias in *P*_gen_ was not observed for VH4Cμ and VH4Cτ.

To investigate the possible effect of this VDJ rearrangement bias on the difference between IgM and IgT in clonotype sharing, we computed the amount of sharing expected for a simulated set of clonotypes from the synthetic repertoire, through the same procedure used to produce Figure S5A. In Figure S7D, we compare these results to the distributions in the experimental data from control fish. For IgM, both the empirical and predicted distributions show similar levels of sharing, except for VH5. In contrast, for IgT, the amount of sharing predicted by the model differs from the experimental data. Hence, comparing the model's and control group's number of clonotypes shared at the level of all 7,000-subsampled clonotypes, the model accounts neither for the high sharing of IgT clonotypes (dark vs. light red), nor for much of the sharing of VH5Cμ clonotypes (Figure S7D). The analysis of the level of sharing of Top50 clonotypes showed the same trend (Figure 3B).

We conclude that the difference in the sharing pattern between IgM and IgT for the Top50 clonotypes in unvaccinated controls is not pre-determined by biases of VDJ rearrangements, but rather reflects selection acting on cells of the B lineage before or at the primary B cell stage.

### Impact of vaccination and boost on the structure of repertoires of most abundant IgM and IgT clonotypes

We next analyzed whether the vaccination or the boost affected the composition of the Top50 IgM and IgT clonotypes.

The distribution of Top50 clonotypes in vaccinated fish (middle column in Figure 2, Figure S5A) was clearly altered in the VH5Cμ repertoire, with a strong increase in the cumulative counts of clonotypes shared by all fish of the vaccinated and boosted groups compared to unvaccinated controls (Figures 2E,F, red and green bars, respectively). A significant proportion of the TCL_Vac_ clonotypes were found in several vaccinated or boosted fish (Figure S5A,e, red and green bars). Interestingly, most of the VH5Cμ clonotypes found in both TCL_Vac_ and TCL_Bst_ (19/26) were not among the Top50 clonotypes from unvaccinated control fish (Figure S5A, Venn diagrams). The change in the VH5Cμ distribution is peculiar because, by contrast, the IgM VH4Cμ and VH8Cμ TCL_Vac_ displayed distributions of sharing similar to those observed in unvaccinated controls. They were mostly found in single vaccinated or boosted fish (Figure S5A,b,h). These results demonstrate that VH5Cμ rearrangements are involved in a public memory response.

The vaccination also altered the distribution of Top50 VH4Cτ clonotypes, that showed a clear increase in the cumulative number of counts for the most abundant clonotypes, from 500 to about 4,500 (Figure 2). However, these expansions were specific to each individual since the cumulative counts of clonotypes shared by several fish remained low (Figure 2). This supports the notion that some VH4Cτ rearrangements are implicated in a private manner in the response to vaccination. As expected, the VH5Cτ and VH9Cτ distributions found in vaccinated and boosted fish were reminiscent of those found in unvaccinated controls with no obvious evidence of vaccine-induced modifications (Figure 2, Figure S5A,n,q, red bars).

Comparison of the cumulative expression of Top50 and Top100 clonotypes (Figure 2, Figure S6A) showed that the increase of shared clonotypes in vaccinated and boosted groups is based on the expansion of the Top50 clonotypes. The particular level of sharing observed for VH5Cμ was very clear even when all 7,000-subsampled clonotypes were analyzed (Figure S6B).

Taken together, these data highlight the contribution of the VH5Cμ to the public response. The responding clonotypes become part of the Top50 clonotypes in the vaccinated and boosted fish, reaching high cumulative counts with a high degree of sharing. The clonotypes implicated in the response were not among the Top50 clonotypes in unvaccinated controls, indicating that the response was recruited from less abundant clonotypes. The analysis of the Top100 and 7,000-subsampled clonotypes supported these conclusions (Figure S6A), and showed that the highly shared clonotypes are mainly found among the most frequent clonotypes.

### Public IgM memory clonotypes of vaccinated fish are frequent in unvaccinated controls, and produced at high frequency by the rearrangement process

**Figure 4:**
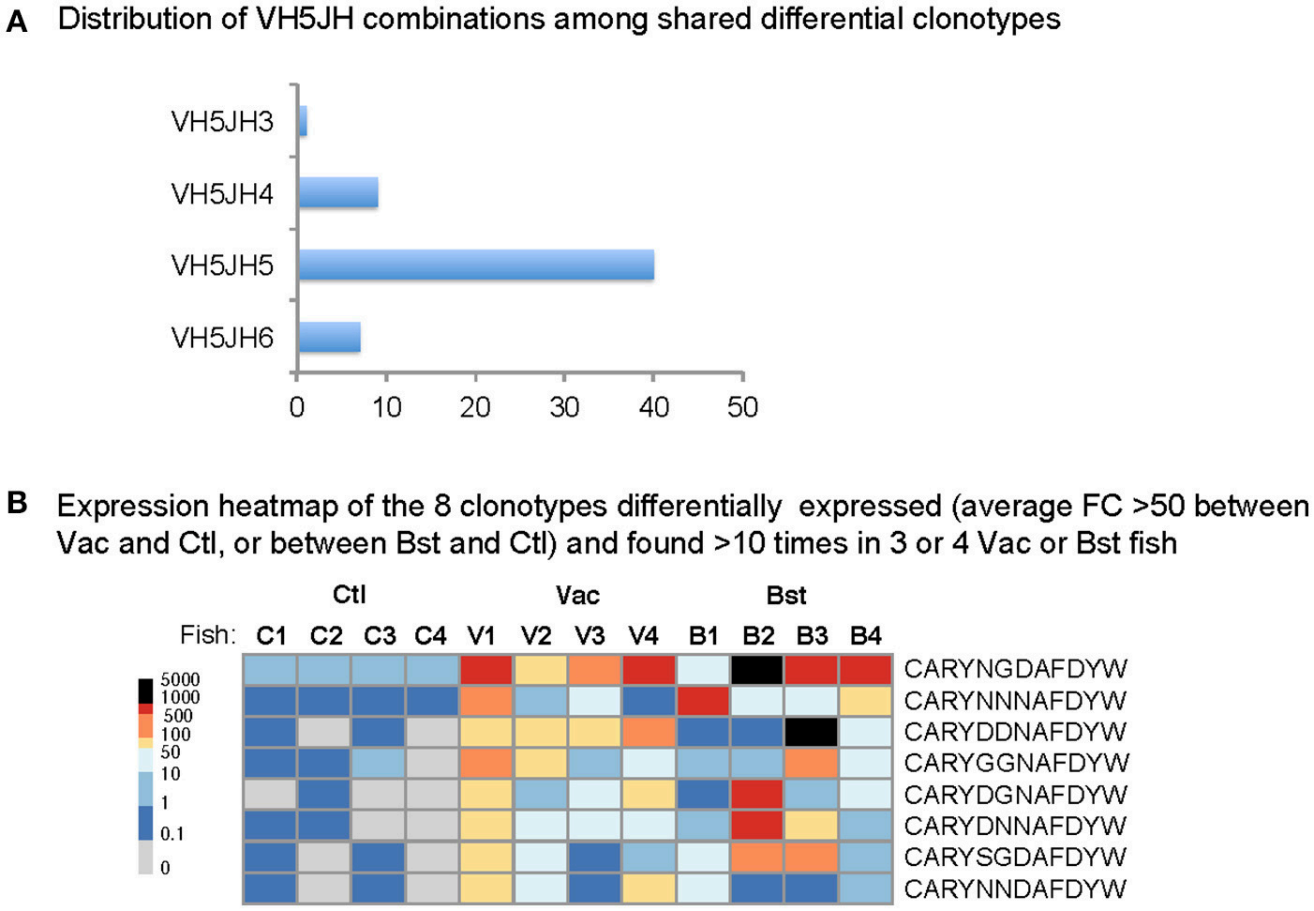
Identification of eight clonotypes of the public response against VHSV. **(A)** The distribution of VH5JH IgM clonotypes shared within vaccinated and boosted groups (detected in ≥3 fish per group) and differentially expressed, compared to the naïve unvaccinated control (Ctl) groups (average FC >50) is shown. **(B)** Expression heatmap of the eight clonotypes detected >10 times in at least vaccinated (vac) or bosted (Bst) fish. All these clonotypes express the VH5JH5 combination. The bar on the left defines the color code of the heatmap, graduated in average MID numbers per subsample.

**Figure 5:**
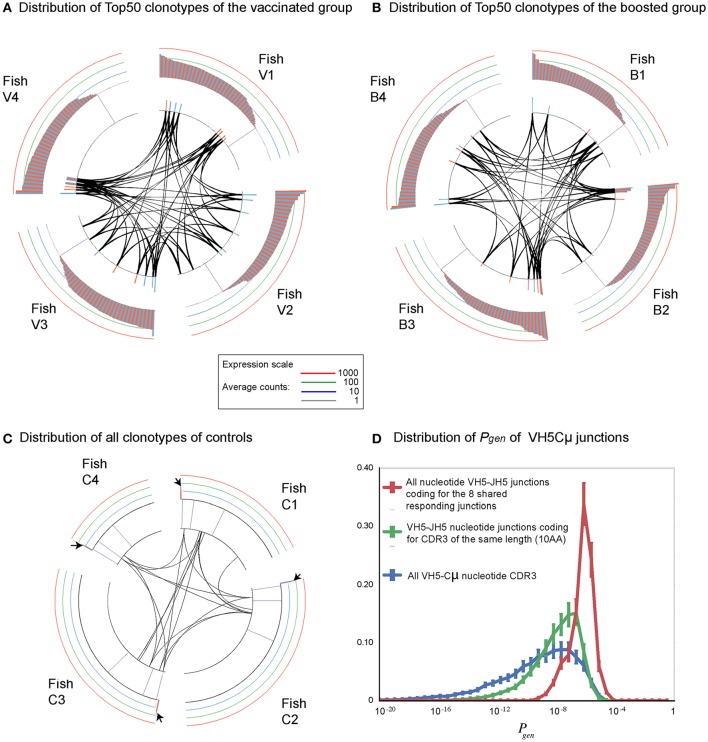
Clonotypes implicated in the shared/public response are already frequent in naive unvaccinated fish. **(A,B)** the eight clonotypes of the VH5 public response are placed into the expression profile of the VH5 TCLvac (respectively TCLbst) of each fish. VH5Cμ Top50 clonotypes of each fish are plotted on circumference of the outer circle, by decreasing expression order, with the alternated red and blue bars representing their respective abundance at logarithmic scale. When detected within the Top50 lists of two individuals, clonotypes are connected with black lines. **(C)** Ranking of the VH5-JH5 “public” clonotypes detected within the whole distribution of VH5Cμ clonotypes in controls. Small arrows denote the most frequent clonotypes at the top side of the distribution **(D)** Distribution of generation probability of junctions coding for the 8 core responding clonotypes (in red), compared with the distribution computed for all VH5Cμ junctions (in blue), or for all VH5JH5 junctions of the same length (in green).

In order to characterize the origin of the VH5Cμ clonotypes implicated in the public memory response to VHSV, we first identified the clonotypes that were (1) detected in at least three out of four fish of the vaccinated or boosted groups, (2) more expressed in vaccinated (or boosted) fish than in unvaccinated, and (3) consistently well-expressed across most fish of the vaccinated and/or boosted groups (i.e., found >10 times in ≥3 fish in Vac or Bst groups; See Supplementary Methods, Table S3). This led us to identify 8 IgM clonotypes. These clonotypes all had the VH5 and JH5 segments and had a common CDR3 length of 10 amino acids (AA), which coincides with the public peak in the spectratypes (Figure 4). Five among these 8 clonotypes had been identified among the most abundant shared clonotypes of the effector response in our previous study of the immediate response following immmunization (18), and differences with the other three sequences were substitutions by AA with similar properties. These 8 public clonotypes were also among the VH5Cμ Top50 clonotype lists from the vaccinated and boosted groups (TCL_Vac_ and TCL_Bst_) (Figures 5A,B, Figures S4C,D).

Each of the these eight public VH5JH5Cμ clonotypes was encoded by 4–17 different nucleotide sequences in vaccinated and boosted fish; this diversity highlights a form of convergence, strongly suggesting that these clonotypes had been subjected to antigen-driven clonal selection during the response (Table S4). Additionally, these Ig sequences had overall very few N/P nucleotides especially at the V-D joint, in line with their increased probability of generation (Figure 5D).

We next examined unvaccinated controls to determine how frequent these clonotypes were, and their degree of sharing prior to vaccination. At least 2 and up to 6 of these clonotypes were detected per control fish in the various sub-samplings. Within the frequency distribution of all the clonotypes found in unvaccinated fish (Figure 5C), they were among the relatively frequent clonotypes.

We then used the computational model presented above to evaluate whether these clonotypes were favored by the VDJ recombination process. Indeed, the distribution of the generation probabilities *P*_gen_ of all the nucleotide sequences coding for the 8 public amino acid clonotypes was higher than for all VH5Cμ nucleotide sequences (Figure 5D). The set of VH5-JH5 rearrangements with a CDR3 length of 10 AA also had a higher probability of generation than the one estimated for all VH5Cμ (Figure 5D). Thus, the high probability of generation of these eight rearrangements is at least partially due to the length of the CDR3 and the usage of JH5.

Collectively, these data suggest that the IgM clonotypes implicated in the public memory response are already present at modestly elevated frequencies in all/most unvaccinated controls, consistently with the higher probability of rearrangement of these clonotypes.

### Distinct VHC combinations differ by their production of similar clonotypes

So far we have considered the public response from the point of view of individual clonotypes taken separately. However, public responses generally involve a mixture of similar clonotypes differing only by one or two “conservative” mutations in their CDR3 i.e., by substitutions with amino acids that share similar properties and usually preserve the recognition of the same epitope (29–31, 18). Such clonotypes—hereafter refered to as “similar”—are often present at variable frequencies in different immunized individuals. Notably, the 8 VH5JH5 clonotypes identified in this study are similar to each other since they differ only by small changes in their CDR3 sequences (Figure 4). For any clonotype found in the public response, a class of “similar” clonotypes may also have responded. The size of these classes in unvaccinated controls is likely to influence the probability that they are shared between distinct individuals and their likelihood of being implicated in a public response. It is therefore important to take into account these classes of “similar” sequences when assessing the propensity of clonotypes to generate a public response.

**Figure 6:**
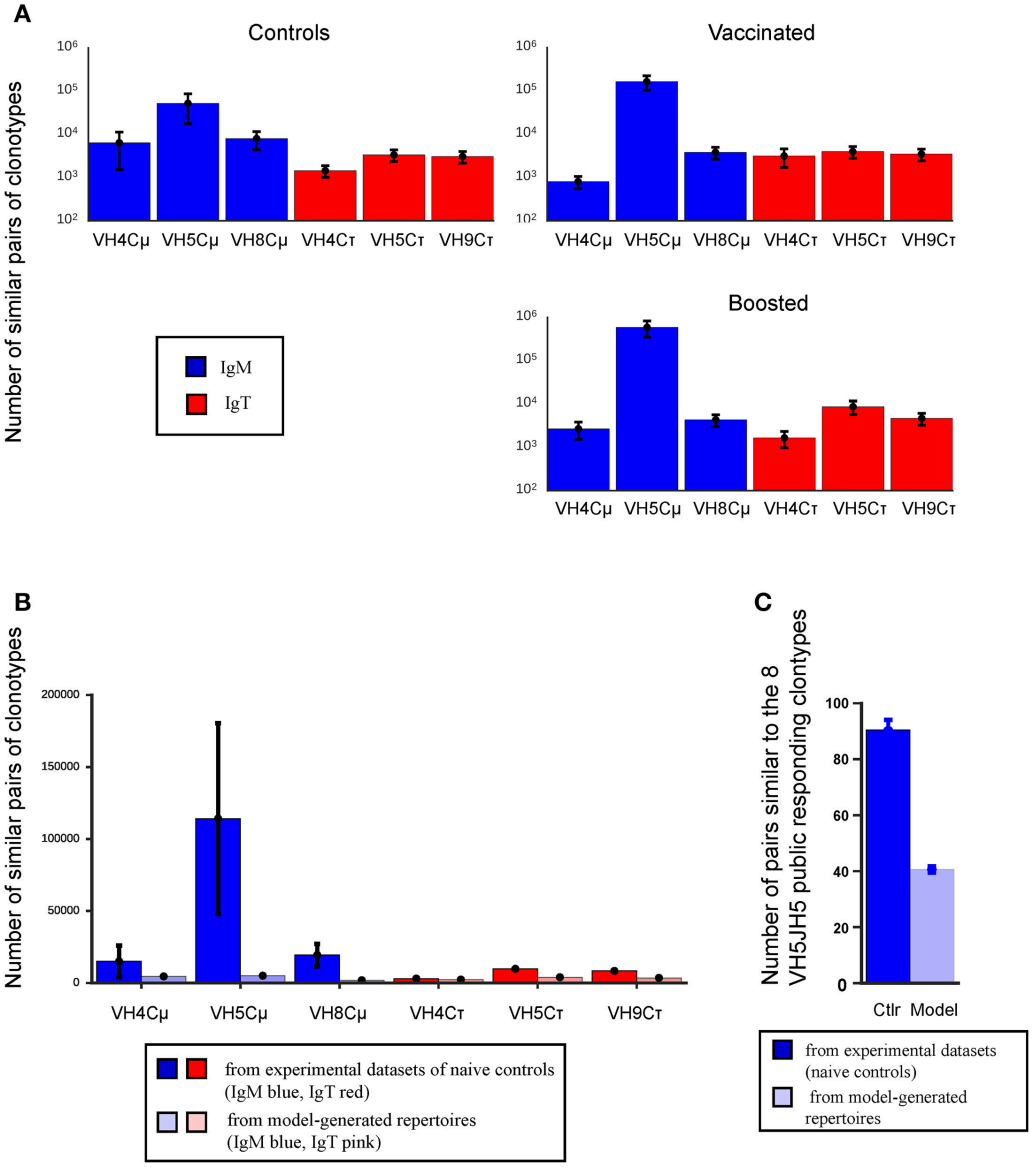
VH5Cμ exhibits distinctly higher CDR3 similarity classes than other VHC, even in naive unvaccinated individuals, but not in the model prediction. Bars show the mean number of similar pairs of clonotypes, (i.e., those with a CDR3 differing by up to 2 AA substitutions within AA groups with similar properties). **(A)** Mean number of pairs of clonotypes deemed similar to each other for each VHC combination and across conditions. For a given condition, pairs of clonotypes were formed from any of the 6 distinct sets of two individual repertoires among the four individuals analyzed. Blue bars represent Cμ, while red bars represent Cτ. Means are calculated across the 6 possible distinct combinations and error bars show their standard error. Note the distinctly larger number of shared clonotype for VH5Cμ. **(B)** Number of similar pairs of clonotypes within the measured control (left) and predicted model (right) repertoires for each VHC combination. Means and standard errors calculated as in **(A)**. Note that the number for VH5Cμ is less distinct compared to **(A)**. **(C)** The number of VH5Cμ clonotypes similar to any of the eight VH5JH5 clonotypes of the public response for the measured control (left bar) and model predicted (right bar) repertoires for the model and three conditions. Means and standard errors are calculated across four individual repertoires.

We first assessed whether distinct VHC combinations differed in the amount of junction similarity exhibited between clonotypes from the same VHC combination. We thus counted pairs of “similar” sequences formed from any set of two individual repertoires among the 4 fish in a given group (i.e., 6 such sets of two repertoires per group), for each VHC combination. This procedure was applied to data obtained from each of the control, vaccinated and boosted conditions (see Figure 6A). We observed that these counts were much higher for VH5Cμ than for any other combination and progressively more in the vaccinated and boosted groups. This feature was present already before vaccination (Figure 6A, left panel). Thus, this higher level of convergence might confer to the VH5Cμ combination a higher probability of generating public responses than the other analyzed VHC combinations.

We then checked whether the computational model could predict this particular feature of the VH5Cμ repertoire. Using the same definition of clonotype similarity class, we assessed the propensity for convergence within repertoires predicted by the recombination model describe earlier, for each VHC combination (see Supplementary Methods), and compared it to the observed data for the unvaccinated controls (Figure 6B). We found that VH5Cμ (along with VH4Cμ) exhibited only slightly higher amounts of convergence than the rest of the combinations in the modeled data (Figure 6B), while it was much higher in the experimental data. The same difference of convergence between model and experimental data was observed when the analysis was restricted to the 8 public clonotypes (Figure 6C), confirming that selection before vaccination (i.e., within the naive repertoire or during the differentiation at earlier stages), rather than the rearrangement process alone, promoted the presence of large shared classes of “similar” clonotypes for the VH5Cμ rearrangement.

## Discussion

High-throughput sequencing has made the complexity of immunological repertoires accessible to experimental investigations (32). We used this approach to characterize the persistent public immune response induced upon vaccination in fish, and to examine its origin in the naïve pre-vaccination repertoire. The public Ab response after vaccination against VHSV, a natural pathogen of rainbow trout, constituted a unique fish model for this approach, with isogenic genetic background available. Our study led us to identify three layers of organization of the IgM repertoire in naïve unvaccinated controls. The first layer comprises the most abundant clonotypes, of which we characterized the top 50 as being mostly present only in given individuals and arising from selection during Ag driven responses. The second layer contains clonotypes favored genetically by the rearrangement process, which are more abundant than if generated via an equally likely rearrangement process. Such clonotypes are more likely to be found in multiple fish, and some of them do give rise to the public response after vaccination, as shown here for the VH5JH5 clonotypes of the anti-VHSV public response. The third layer, which we did not characterize in detail, is made of less frequent clonotypes and encompasses the main fraction of the repertoire. Collectively, our findings identify two complementary processes of the naïve repertoire that are associated with the VH5 public memory response: (1) the high probability of rearrangements encoding public junctions, and (2) a high frequency of selection of classes of clonotypes with CDR3 similar to each other into the naive pre-vaccination VH5 IgM repertoire.

Importantly, while we have no biochemical evidence that the 8 public clonotypes encode Ab directed against the VHSV epitope, this specificity is strongly suggested by several lines of evidence: following our definition of “public,” the set of 8 clonotypes was expanded in all immunized individuals, as in our previous study. These clonotypes or similar ones were not expanded in isogenic trout after vaccination against another virus, the Infectious Pancreatic Necrosis Virus (data not shown). Second, these expanded clonotypes were encoded by multiple nucleotide sequences, which is a good indication for Ag driven selection. Altogether, these observations indicate that the 8 public sequences are very different from clonotypes named “public” in other contexts. Indeed, another definition for public clonotypes that has recently been used by different authors is “shared by at least two individuals” (17, 33), or (3) for T cells.

### Contrasted structures of IgM and IgT repertoires in spleen before vaccination

**Figure 7:**
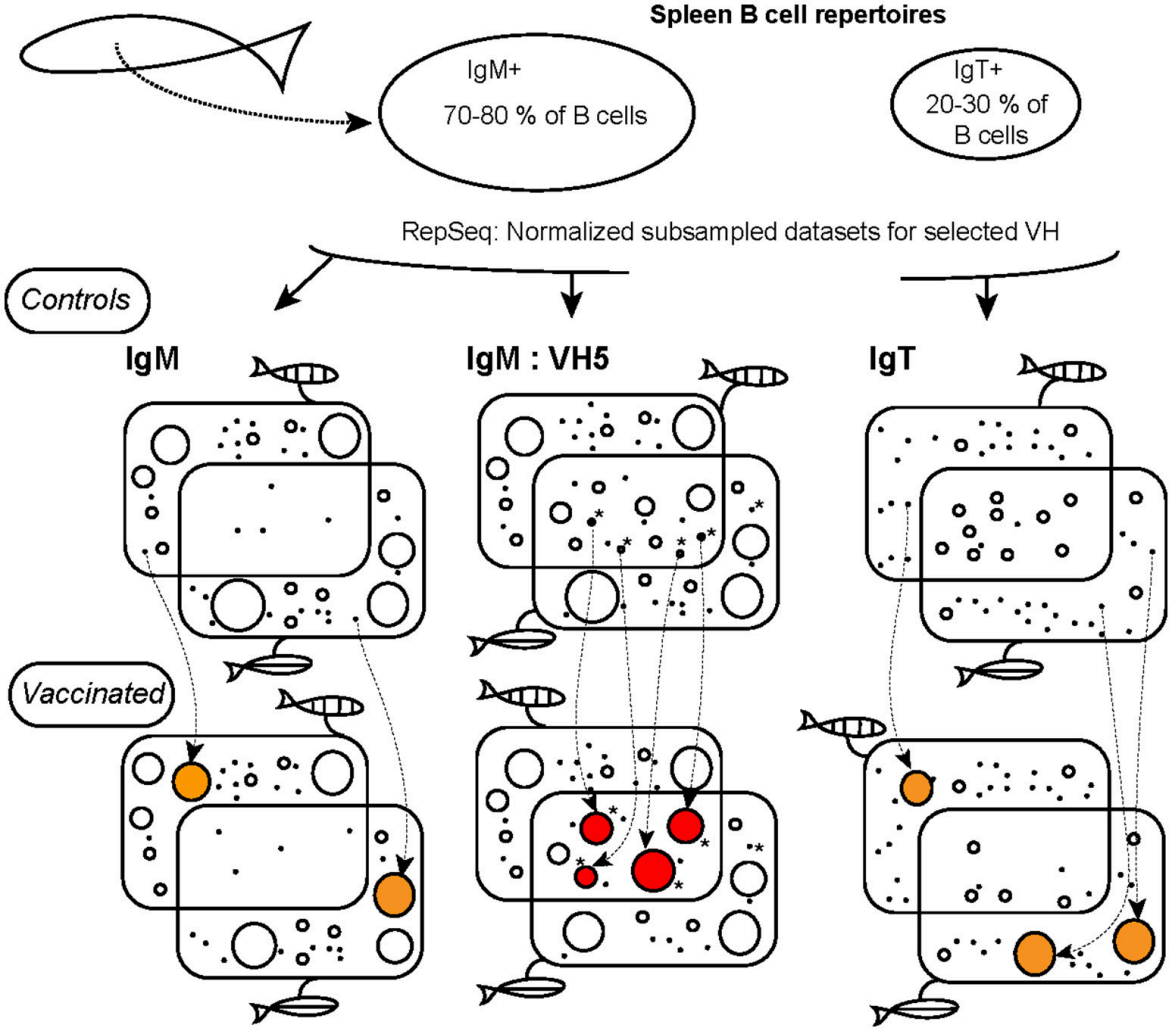
Schematic model of trout IgM and IgT responses against the virus. The diameter of circles reflects the size of clonotypes. Two individual fish are identified by vertical or horizontal stripes. Naïve unvaccinated IgT repertoires contain shared clonotypes of small size, even the Top clonotypes, while IgM repertoires included clonotypes of large size that are mainly specific to individuals. Response to the virus comprises private IgM or IgT clonotypes (represented in orange), or public clonotypes (represented in red). Note that IgT private response reduces the level of sharing of top clonotypes, and leads to highly frequent clonotypes specific to the virus. The VH5 IgM public response is based on highly shared clonotypes already present in naïve unvaccinated controls. Sets of clonotypes similar (but not identical) to public anti VHSV clonotypes, are denoted by^*^. Such sets of similar clonotypes are very prominent in the VH5 IgM repertoire. The proportions of clonotypes of different sizes reflect the global structure of the repertoires, but does not exactly correspond to the average distribution of our datasets (see Figure S4 for such a representation).

For both IgM and IgT, most VDJ combinations were present at low frequency in unvaccinated controls. However, the normalized distributions of IgM and IgT clonotypes differed when considering the most abundant ones. The abundance of Top50 clonotypes was typically 10–20 times higher for IgM than for IgT. This pattern was not predicted by our computational model of rearrangements, indicating that it was due to post-recombination selective processes exerted on B cells before vaccination. The spleen IgM and IgT repertoires also differed in the degree of sharing of the most frequent clonotypes (Figure 7). Very few top IgM clonotypes, except for VH5, were shared by more than two individuals, while more than one third of the top IgT clonotypes were shared. A similar pattern was observed when considering all clonotypes. Hence, IgM most abundant clonotypes may reflect each individual's unique immunological history, while the stereotyped IgT repertoire may be shaped by common components of the environment. Since IgT is the main isotype involved in mucosal immunity, this may reflect the effect of common components of the microbiota, in line with the key role of intestinal microbes in the generation of the IgA repertoire in human and mice (34). Additionally, differences of IgH mRNA expression level between (activated) IgM^+^ and IgT^+^ B cells may also affect clonotype sharing in sequence data. This remains difficult to take into account because these expression levels are poorly defined.

### Fish maintain IgM and IgT components of the response to vaccination over 5 months in the spleen

The B cell repertoire response signatures persisted in spleen for months after vaccination, involving public clonotypes previously found in fish infected twice at 3 weeks interval (18). Our previous data suggested that the most frequent clonotypes of the VH5Cμ public response after two infections in quick succession could represent up to 25–30% of the total VH5Cμ transcripts (18). In this dataset, they reached 10–15% at most, suggesting that the size of public VHSV-specific top clonotypes is smaller 5 months post-vaccination. This is consistent with the maintenance of a lower pool of “memory” clonotypes after the elimination of the attenuated virus. This result differs from immunizations with hapten-carrier conjugates (TNP-KLH), where the high-affinity Abs appeared only 3 months after trout immunization (35). In line with the notion that the vaccination induced the generation of memory cells, the boost was associated with a marked accumulation of virus-specific antibody-secreting cells in the pronephros, and an increased abundance of similar clonotypes shared by all fish in the spleen for the VH5Cμ rearrangement that contained the public response (Figure 2).

### The VH repertoire involved in public memory responses has distinctive characteristics

Eight VH5JH5 clonotypes were expanded in most or all vaccinated fish, with CDR3 differing only by one or two “conservative” substitutions. Overall, this VH5JH5 IgM response can therefore be named truly “public” (36). These public clonotypes were among the most frequent in VH5Cμ, and were as frequent as the top clonotypes of the other responding VHC families (data not shown), indicating that public and private responses were of the same order of magnitude.

Public responses depend on clones that are relatively frequent before immunization (5, 16, 17, 37). It is more likely for such clones to be selected by their specific epitope early in the response in all individuals, and to outcompete other clones as was proposed for T-cell receptors (38). Most public VH5JH5Cμ clonotypes were detected in several unvaccinated controls, and ranked on the high side of the frequency distribution, although far below the Top50. Our computational model showed that these public rearrangements were more likely produced by the recombination machinery than other VH5-JH5 junctions. The IgM public response is therefore at least partly biased by recombination. In contrast, the clonotypes involved in the large private VH4Cτ expansions observed in infected fish were not detected in controls (data not shown). These findings should be put in the context of a growing evidence for repertoire biasing across vertebrates. In adult zebrafish, Weinstein et al. found that 250 clonotypes were shared much more often than predicted by a uniform random model (2). A strong bias in the recombination process was also reported for human (10) and mouse (17) B cell repertoires, and the effect of such bias on the sharing of repertoires has been extensively studied for T cell receptors (39–42).

In human and mice, public responses comprise multiple clonotypes with the same VH and JH, and CDR3 amino acid sequences of identical length differing by one or two AA (9, 26–28, 38, 39). To investigate the role of this property in our system, we first determined the fraction of the repertoire that consisted in classes of similar clonotypes differing from each other in such a way. We found that this fraction was higher for VH5Cμ than for any other VHC combination even in unvaccinated controls, suggesting a generic characteristic of the VH5Cμ combination independent of the public response to VHSV. Since our model of rearrangements did not predict this distinctive property of VH5Cμ (Figure 6), it must be mainly due to the selection processes shaping the naive pre-vaccination repertoire, possibly involving previous exposure to environmental microbes, as reported for mice (17). Thus, the junctions of the public clonotypes are not only favored by the rearrangement process, but also are found together with many other highly similar VH5DHJH5 junctions in unvaccinated controls that may bind related epitopes. A schematic representation of the IgM public response, compared to IgM and IgT private responses, is shown in Figure 7. VHSV being a natural pathogen of rainbow trout evolving as a viral quasi-species, our data may suggest that some properties of the VH5Cμ repertoire have been selected during trout/VHSV co-evolution. Further studies in different host/pathogens models across vertebrates will determine whether the degree of convergence of IgH CDR3 between different VH (before immunization) is correlated to their implication in public responses.

## Conclusion

Our data provide novel insights into the generation of public memory responses in the context of vaccination with an attenuated virus in fish. This information could be useful practically: if public clonotype expansions are correlated to protective specific Ab responses against pathogens, they could constitute excellent biomarkers of the efficiency of vaccines in aquaculture. On the other hand, observations from fish have a further interest when compared to other vertebrates. Teleost fish are an old, basal lineage of vertebrates, which has evolved in parallel with tetrapods for more than 350 million years. Their immune system shares basic components with other vertebrates, but is nonetheless very divergent. Regarding B cells and Ab responses, lack of lymph nodes and germinal centers, slow kinetics of Abs responses, poor affinity maturation are important differences compared to humans and mice. Hence, the fish B cell system is adapted to very different anatomical and physiological constraints (43). Therefore, features conserved with respect to the common ancestors of fish and mammals are likely to be essential, while other characteristics may represent fish-specific original solutions. The implication of clonotypes with a high probability of generation in public responses therefore appears to be one of the fundamental traits conserved across vertebrates, for B and T cells (39, 41). This mechanism could have been selected during evolution because it favored B cells mediating public responses against microbial epitopes. In contrast, the idea that for certain VH (VH5 in this study) large classes of “similar” clonotypes are positively selected before vaccination, guaranteeing the presence of relevant clonotypes targeting key epitopes, remains to be tested in other groups of vertebrates. Overal, our data show that the fish public memory antibody response to a virus is determined at three levels: by recombination biases, by selection acting on the formation of the pre-vaccination repertoire, and by convergent selection of functionally similar clonotypes during the response. Our data pave the way for a better definition of the features of public responses across vertebrates.

## Author contributions

SuM, TM, AW, SF, and PB conceived the project. SuM, AS, OS, SF, and PB designed experiments. SuM and PB performed wet-lab experiments. MPT, AW,TM designed the computational model. SuM, LJ, MPT, SM, WC, AS, FC, AW, TM, and PB performed primary data analysis. AS and EQ provided resources. SuM, MPT, TM, AW, SF, and PB wrote the manuscript. All authors edited the manuscript.

### Conflict of interest statement

The authors declare that the research was conducted in the absence of any commercial or financial relationships that could be construed as a potential conflict of interest. The reviewer YL declared a past co-authorship with several of the authors (MT, TM, AW) to the handling Editor.

## References

[1] Jerne N What precedes clonal selection? ontogeny of acquiredimmunity. In: Proceedings of the CIBA Foundation Symposium (Amsterdam: CIBA) (1971).

[2] Weinstein JA Jiang N White RA Fisher DS Quake SRS White III R. High-throughput sequencing of the zebrafish antibody repertoire. Science (2009) 324:807–11. 10.1126/science.1170020 19423829 PMC3086368

[3] Covacu R Philip H Jaronen M Almeida J Kenison JE Darko S. System-wide analysis of the T cell response. Cell Rep. (2016) 14:2733–44. 10.1016/j.celrep.2016.02.056 26972015 PMC4805488

[4] Ma KY He C Wendel BS Williams CM Xiao J Yang H. Immune repertoire sequencing using molecular identifiers enables accurate clonality discovery and clone size quantification. Front Immunol. (2018) 9:33. 10.3389/fimmu.2018.00033 29467754 PMC5808239

[5] Mora T Walczak AM Bialek W Callan CG. Maximum entropy models for antibody diversity. Proc Natl Acad Sci USA. (2010) 107:5405–10. 10.1073/pnas.1001705107 20212159 PMC2851784

[6] Galson JD Truck J Fowler A Munz M Cerundolo V Pollard AJ. In-depth assessment of within-individual and inter-individual variation in the B cell receptor repertoire. Front Immunol. (2015) 6:531. 10.3389/fimmu.2015.00531 26528292 PMC4601265

[7] DeWitt WS Lindau P Snyder TM Sherwood AM Vignali M Carlson CS. A public database of memory and naive B-cell receptor sequences. PLoS ONE (2016) 11:e0160853. 10.1371/journal.pone.0160853 27513338 PMC4981401

[8] Krasnov A Jorgensen SM Afanasyev S. Ig-seq: deep sequencing of the variable region of atlantic salmon IgM heavy chain transcripts. Mol Immunol. (2017) 88:99–105. 10.1016/j.molimm.2017.06.022 28623734

[9] Fu X Sun J Tan E Shimizu K Reza MS Watabe S. High-throughput sequencing of the expressed torafugu (*Takifugu rubripes*) antibody sequences distinguishes IgM and IgT repertoires and reveals evidence of convergent evolution. Front Immunol. (2018) 9:251. 10.3389/fimmu.2018.00251 29515575 PMC5826340

[10] Elhanati Y Sethna Z Marcou Q Callan CG Jr Mora T Walczak AM. Inferring processes underlying B-cell repertoire diversity. Philos Trans R Soc Lond B Biol Sci. (2015) 370:20140243. 10.1098/rstb.2014.0243 26194757 PMC4528420

[11] Vollmers C Sit RV Weinstein JA Dekker CL Quake SR. Genetic measurement of memory B-cell recall using antibody repertoire sequencing. Proc Natl Acad Sci USA. (2013) 110:13463–8. 10.1073/pnas.1312146110 23898164 PMC3746854

[12] Jackson KJ Liu Y Roskin KM Glanville J Hoh RA Seo K. Human responses to influenza vaccination show seroconversion signatures and convergent antibody rearrangements. Cell Host Microbe (2014) 16:105–14. 10.1016/j.chom.2014.05.013 24981332 PMC4158033

[13] Acha-Orbea H Mitchell DJ Timmermann L Wraith DC Tausch GS Waldor MK. Limited heterogeneity of T cell receptors from lymphocytes mediating autoimmune encephalomyelitis allows specific immune intervention. Cell (1988) 54:263–73. 10.1016/0092-8674(88)90558-2 2455603

[14] Briles DE Forman C Hudak S Claflin JL. Anti-phosphorylcholine antibodies of the T15 idiotype are optimally protective against *Streptococcus pneumoniae*. J Exp Med. (1982) 156:1177–85. 10.1084/jem.156.4.1177 7153709 PMC2186814

[15] Mi QS Zhou L Schulze DH Fischer RT Lustig A Rezanka LJ. Highly reduced protection against *Streptococcus pneumoniae* after deletion of a single heavy chain gene in mouse. Proc Natl Acad Sci USA. (2000) 97:6031–6. 10.1073/pnas.110039497 10811914 PMC18553

[16] Wang C Liu Y Cavanagh MM Le Saux S Qi Q Roskin KM. B-cell repertoire responses to varicella-zoster vaccination in human identical twins. Proc Natl Acad Sci USA. (2015) 112:500–5. 10.1073/pnas.1415875112 25535378 PMC4299233

[17] Greiff V Menzel U Miho E Weber C Riedel R Cook S. Systems analysis reveals high genetic and antigen-driven predetermination of antibody repertoires throughout B cell development. Cell Rep. (2017) 19:1467–78. 10.1016/j.celrep.2017.04.054 28514665

[18] Castro R Jouneau L Pham HP Bouchez O Giudicelli V Lefranc MP. Teleost fish mount complex clonal IgM and IgT responses in spleen upon systemic viral infection. PLoS Pathog. (2013) 9:e1003098. 10.1371/journal.ppat.1003098 23326228 PMC3542120

[19] Hansen J Landis E Phillips R. Discovery of a unique Ig heavy-chain isotype (IgT) in rainbow trout: implications for a distinctive B cell developmental pathway in teleost fish. Proc Natl Acad Sci USA. (2005) 102:6919–24. 10.1073/pnas.0500027102 15863615 PMC1100771

[20] Salinas I Zhang Y-A Sunyer JO. Mucosal immunoglobulins and B cells of teleost fish. Dev. Comp. Immunol. (2011) 35:1346–65. 10.1016/j.dci.2011.11.009 22133710 PMC3428141

[21] Zhang Y-A Salinas I Li J Parra D Bjork S Xu Z. IgT, a primitive immunoglobulin class specialized in mucosal immunity. Nat. Immunol. (2010) 11:827–35. 10.1038/ni.1913 20676094 PMC3459821

[22] Bromage ES Kaattari IM Zwollo P Kaattari SL. Plasmablast and plasma cell production and distribution in trout immune tissues. J Immunol. (2004) 173:7317–23. 10.4049/jimmunol.173.12.7317 15585855

[23] Marcou Q Mora T Walczak AM. High-throughput immune repertoire analysis with IGoR. Nat Commun. (2018) 9:561–9. 10.1038/s41467-018-02832-w 29422654 PMC5805751

[24] Ma C Ye J Kaattari SL. Differential compartmentalization of memory B cells versus plasma cells in salmonid fish. Eur J Immunol. (2013) 43:360–70. 10.1002/eji.201242570 23255215

[25] Farber DL Netea MG Radbruch A Rajewsky K Zinkernagel RM. Immunological memory: lessons from the past and a look to the future. Nat Rev Immunol. (2016) 16:124–8. 10.1038/nri.2016.13 26831526

[26] Cover TM Thomas JA Elements of Information Theory. Hoboken, NJ: Wiley (2006).

[27] Mora T Walczak AM. Renyi entropy, abundance distribution, and the equivalence of ensembles. Phys Rev E (2016) 93:052418. 10.1103/PhysRevE.93.052418 27300934

[28] Murugan A Mora T Walczak AM Callan CG. Statistical inference of the generation probability of T-cell receptors from sequence repertoires. Proc Natl Acad Sci USA. (2012) 109:16161–6. 10.1073/pnas.1212755109 22988065 PMC3479580

[29] Cibotti R Cabaniols JP Pannetier C Delarbre C Vergnon I Kanellopoulos JM. Public and private V beta T cell receptor repertoires against hen egg white lysozyme (HEL) in nontransgenic versus HEL transgenic mice. J Exp Med. (1994) 180:861–72. 10.1084/jem.180.3.861 8064237 PMC2191659

[30] Bousso P Casrouge A Altman JD Haury M Kanellopoulos J Abastado J-PP. Individual variations in the murine T cell response to a specific peptide reflect variability in naive repertoires. Immunity (1998) 9:169–78. 10.1016/S1074-7613(00)80599-3 9729037

[31] Lin MY Welsh RM. Stability and diversity of T cell receptor repertoire usage during lymphocytic choriomeningitis virus infection of mice. J Exp Med. (1998) 188:1993–2005. 10.1084/jem.188.11.1993 9841914 PMC2212379

[32] Friedensohn S Khan TA Reddy ST. Advanced methodologies in high-throughput sequencing of immune repertoires. Trends Biotechnol. (2017) 35:203–14. 10.1016/j.tibtech.2016.09.010 28341036

[33] Greiff V Weber CR Palme J Bodenhofer U Miho E Menzel U. Learning the high-dimensional immunogenomic features that predict public and private antibody repertoires. J Immunol. (2017) 199:2985–97. 10.4049/jimmunol.1700594 28924003

[34] Macpherson AJ Koller Y McCoy KD. The bilateral responsiveness between intestinal microbes and IgA. Trends Immunol. (2015) 36:460–70. 10.1016/j.it.2015.06.006 26169256

[35] Ye J Kaattari IM Kaattari SL. The differential dynamics of antibody subpopulation expression during affinity maturation in a teleost. Fish Shellfish Immunol. (2011) 30:372–7. 10.1016/j.fsi.2010.11.013 21093593

[36] Castro R Navelsaker S Krasnov A Du Pasquier L Boudinot P. Describing the diversity of Ag specific receptors in vertebrates: contribution of repertoire deep sequencing. Dev Comp Immunol. (2017) 75:28–37. 10.1016/j.dci.2017.02.018 28259700

[37] Sigal NH Gearhart PJ Klinman NR. The frequency of phosphorylcholine-specific B cells in conventional and germfree BALB/C mice. J Immunol. (1975) 114:1354–8. 1078833

[38] Bousso P Levraud JP Kourilsky P Abastado JP. The composition of a primary T cell response is largely determined by the timing of recruitment of individual T cell clones. J Exp Med. (1999) 189:1591–600. 10.1084/jem.189.10.1591 10330438 PMC2193643

[39] Venturi V Kedzierska K Price DA Doherty PC Douek DC Turner SJ. Sharing of T cell receptors in antigen-specific responses is driven by convergent recombination. Proc Natl Acad Sci USA. (2006) 103:18691–6. 10.1073/pnas.0608907103 17130450 PMC1693724

[40] Elhanati Y Murugan A Callan CGJr Mora T Walczak AM. Quantifying selection in immune receptor repertoires. Proc Natl Acad Sci USA. (2014) 111:9875–80. 10.1073/pnas.1409572111 24941953 PMC4103359

[41] Madi A Shifrut E Reich-Zeliger S Gal H Best K Ndifon W. T-cell receptor repertoires share a restricted set of public and abundant CDR3 sequences that are associated with self-related immunity. Genome Res. (2014) 24:1603–12. 10.1101/gr.170753.113 25024161 PMC4199372

[42] Madi A Poran A Shifrut E Reich-Zeliger S Greenstein E Zaretsky I. T cell receptor repertoires of mice and humans are clustered in similarity networks around conserved public CDR3 sequences. Elife (2017) 6:e22057. 10.7554/eLife.22057 28731407 PMC5553937

[43] Magadan S Sunyer OJ Boudinot P. Unique features of fish immune repertoires: particularities of adaptive immunity within the largest group of vertebrates. Results Probl Cell Differ. (2015) 57:235–64. 10.1007/978-3-319-20819-0_10 26537384 PMC5124013

