# Supplementary material for: Origin of Public Memory B Cell Clones in Fish After Antiviral Vaccination
Source: Front Immunol. 2018 Sep 27;9:2115. doi: 10.3389/fimmu.2018.02115 (PMC6170628; doi:10.3389/fimmu.2018.02115)
Supplement: Supplementary file 1 [file Table_1.pdf]

**Table S1. Neutralising Ab titers and anti-VHSV ELISPOT assays.**

A. Neutralising antibody titers in vaccinated (V) and boosted (B) fish

| | | | | | | |
|---|---|---|---|---|---|---|
| Vaccinated group | V1 | V2 | V3 | V4 | V5 | V6 |
| | 1/10 000 | 1/4000 | 1/10 000 | 1/10 000 | 1/10 000 | 1/4000 |
| Boosted group | B1 | B2 | B3 | B4 | B5 | B6 |
| | 1/4000 | 1/10 000 | 1/10 000 | 1/4000 | 1/10 000 | 1/10 000 |

B. Numbers of specific IgM producing B cells per million pronephros leukocytes in vaccinated (V) and in boosted (B) fish

| | | | | | | |
|---|---|---|---|---|---|---|
| Vaccinated group | V1 | V2 | V3 | V4 | V5 | V6 |
| | 0.8 | 0.73 | 1.07 | 0.27 | 0.47 | 0.53 |
| Boosted group | B1 | B2 | B3 | B4 | B5 | B6 |
| | 12.7 | 26.1 | 33.6 | 14.3 | 18.5 | 32.3 |
