# Supplementary material for: Origin of Public Memory B Cell Clones in Fish After Antiviral Vaccination
Source: Front Immunol. 2018 Sep 27;9:2115. doi: 10.3389/fimmu.2018.02115 (PMC6170628; doi:10.3389/fimmu.2018.02115)
Supplement: Supplementary file 2 [file Table_2.pdf]

Table S2. Clonotype diversity in controls, for each VH-C combination

A. Number of distinct clonotypes detected in controls for each VH-C combination

{V;C} /Fish	Ctrl group Ctrl1	Ctrl2	Ctrl3	Ctrl4	All Ctrl
VH4.1;Cμ	1857.3[1]	2278.7	2068.9	1584.2	7705.2
VH5.1;Cμ	1121.9	1504	1456.2	820	4506.8
VH8.1;Cμ	1281.1	2165	1773.1	1714.2	6732.1
VH4.1;Cτ	6379.8	6428.3	6436.3	6367.2	24854.4
VH5.4;Cτ	6098.2	6298.4	6340.7	6182.6	23442.2
VH9.2;Cτ	6489	6513.9	6565.1	6339.5	24681.7

[1] Average values over 10 subsamplings of 7000 MID, per VHC combination and individual fish

B. Number of HS/public clonotype per 1000 clonotypes in Controls (Ctl), in vaccinated fish (Vac) and in challenged fish (Cha).

{V;C} group	Ctl HS[1]	Ctl Public[2]	Vac HS	Vac Public	Bst HS	Bst Public
VH4.1;Cμ	0,8[3]	0,026	1,1	0,073	1,6	0,092
VH5.1;Cμ	17,0	5,0	21,6	6,8	26,8	8,7
VH8.1;Cμ	3,9	0,5	4,7	0,9	6,5	1,2
VH4.1;Cτ	5,0	1,2	3,1	0,5	6,4	1,0
VH5.4;Cτ	12,4	3,8	10,8	2,9	16,5	5,3
VH9.2;Cτ	9,2	2,7	9,2	2,8	10,6	3,4

[1]HS: highly shared clonotypes found in at least 3 fish per group
[2] Public clonotypes are found in all fish within a group
[3] Data are average values from 10 subsamplings of 7000 MID per individual.

C. Entropy of rearrangement sets generated by the computational model for each VH-C combination

VH/C combination	Entropy (bits)
VH4/Cμ	33.0
VH5/Cμ	30.2
VH8/Cμ	33.9
VH4/Cτ	35.6
VH5/Cτ	33.9
VH9/Cτ	34.9

Although diversity estimates based on entropies are more robust than those estimated from observed number of species (24), note that uncertainty in these estimates is exponentiated when they are mapped back to an estimate in raw numbers.
