# Supplementary material for: Origin of Public Memory B Cell Clones in Fish After Antiviral Vaccination
Source: Front Immunol. 2018 Sep 27;9:2115. doi: 10.3389/fimmu.2018.02115 (PMC6170628; doi:10.3389/fimmu.2018.02115)
Supplement: Supplementary file 3 [file Table_3.pdf]

Table S3. Responding shared clonotypes.

Numbers of shared clonotypes found >10 times in >3fish per immunized group, for FC threshold 50, 25 and 10 between Vac (or Bst) group and controls. For a threshold of k=50, only eight "core" responding IgM clonotypes, all expressing VH5-JH5 rearrangements, were counted in average more than 10 times per subsample in at least three fish of Vaccinated or Boosted groups At lower fold-change thresholds and at lower levels of expression across vaccinated and boosted fish, several IgM clonotypes and even a few IgT clonotypes were consistently increased in most individuals after immunization. This might also contribute to the shared response against the virus.

		FC(Vac/Ctl) >=50	FC(Bst/Ctl) >=50	FC(Vac/Ctl) >=25	FC(Bst/Ctl)> =25	FC(Vac/Ctl) >=10	FC(Bst/Ctl) >=10
		Number of clonotypes found >10 times in >=3 fish in Vac or Bst	Number of clonotypes found >10 times in >=3 fish in Vac or Bst	Number of clonotypes found >10 times in >=3 fish in Vac or Bst	Number of clonotypes found >10 times in >=3 fish in Vac or Bst	Number of clonotypes found >10 times in >=3 fish in Vac o	Number of clonotypes found >10 times in >=3 fish in Vac or Bst
{V;C} group	JH						
VH4.1;Cμ	all	0	0	0	0	0	0
VH5.1;Cμ	JH3	0	0	0	0	0	0
	JH4	0	0	0	1[4]	1[6]	1[8]
	JH5	7[1]	8[2]	10[3]	8[5]	10[7]	9[9]
	JH6	0	0	0	0	0	0
VH8.1;Cμ	all	0	0	0	0	0	0
VH4.1;Cτ	all	0	0	0	0	0	0
VH5.4;Cτ	all	0	0	0	0	0	0
VH9.2;Cτ	all	0	0	0	0	0	0

[1]. CARYDDNAFDYW, CARYDGNAFDYW, CARYDNNAFDYW, CARYGGNAFDYW, CARYNGDAFDYW, CARYNNDAFDYW, CARYSGDAFDYW [2]. CARYDDNAFDYW, CARYDNNAFDYW, CARYGGNAFDYW, CARYNGDAFDYW, CARYNNDAFDYW, CARYNNNAFDYW, CARYDGNAFDYW, CARYSGDAFDYW [3] CARYDDNAFDYW, CARYDGNAFDYW, CARYDNNAFDYW, CARYGGNAFDYW, CARYGGYAFDYW, CARYNGDAFDYW, CARYNNDAFDYW, CARYNNNAFDYW, CARYSGDAFDYW, CARYTGYAFDYW; [4] CARYATAYFDYW; [5] CARYATAYFDYW, CARYDDNAFDYW, CARYDGNAFDYW, CARYDNNAFDYW, CARYGGNAFDYW, CARYNGDAFDYW, CARYNNDAFDYW, CARYSGDAFDYW; [6] CARENYNYFDYW; [7] CARYNGDAFDYW, CARYNNNAFDYW, CARYTGYAFDYW, CARYSGDAFDYW, CARYDDNAFDYW, CARYDNNAFDYW, CARYDGNAFDYW, CARYGGNAFDYW, CARYGGYAFDYW, CARYNNDAFDYW, [8]CARYATAYFDYW; [9] CARYNGDAFDYW, CARYNNNAFDYW, CARYTGYAFDYW, CARYSGDAFDYW, CARYDDNAFDYW, CARYDNNAFDYW, CARYDGNAFDYW, CARYGGNAFDYW, CARYNNDAFDYW
