# Supplementary material for: Origin of Public Memory B Cell Clones in Fish After Antiviral Vaccination
Source: Front Immunol. 2018 Sep 27;9:2115. doi: 10.3389/fimmu.2018.02115 (PMC6170628; doi:10.3389/fimmu.2018.02115)
Supplement: Supplementary file 4 [file Table_4.pdf]

Table S4. Multiple nucleotide CDR3 sequences coding for the eight AA sequences of core shared responding clonotypes

To filter sequences likely due to sequencing/PCR errors, we consider only the most frequent, accounting to 99% of counts for each AA junction. AACDR3 were encoded by 4 to 17 distinct nucleotide sequences. DH4, 5, 7 and 8 were identified in these sequences, and their contribution to CDR3 was up to 9 nucleotides. Almost all sequences apparently contained N diversity, but essentially at the D/J junction. Most differences between nucleotide sequences encoding a given AA CDR3 were due to N diversity or differential DH/JH trimming, although variability in the VH and JH sequences was significant for CARYDNNAFDYW in particular. Since fish are double haploid clones, such differences should be due to hypermutation (or might partly come from highly similar duplicated gene segments). Remarkably, almost no N/P diversity was observed at the V/D joint, which likely contributes to the high probability of generation of these rearrangements.

A. Numbers of nucleotide junction sequences coding 99% of MID for the eight AA sequences of core shared responding clonotypes

	Nb of nt junctions coding 99% in Vaccinated fish (in common with Challenged)	Nb of nt junctions coding 99% in Challenged fish
CARYNGDAFDYW	9 (7)	7
CARYNNNAFDYW	7(3)	5
CARYDDNAFDYW	3(3)	4
CARYGGNAFDYW	10(7)	12
CARYDGNAFDYW	10(4)	6
CARYDNNAFDYW	9(5)	10
CARYSGDAFDYW	11(5)	17
CARYNNDAFDYW	7(2)	5

B. Detailed analysis of nucleotide CDR3 sequences

CARYNGDAFDYW

VH5			JH5
tgtgcccggtaca	DH or N/P diversity		acaatgcttttgactactgg
	DH4: ataacaacggggg		
	DH5: ccatatagcggggt		
	DH7: cagaataacggc		
	DH8: tacactgggagc		
tgtgcccggta	**taacggc**	*g* *(D7)*[1]	atgcttttgactactgg
tgtgcccggta	**taacgg**	*ag* *(D7)*[2]	atgcttttgactactgg
tgtgcccggt	**acaacgg**	*cg* *(D4)*	atgcttttgactactgg
tgtgcccggt	**acaacgggg**	*(D4)*	atgcttttgactactgg
tgtgcccggta	**taacggc**	*g* *(D7)*	atgcctttgactactgg
tgcgcccggta	**taacggc**	*g* *(D7)*	atgcttttgactactgg
tgtgcccggta	**taacgg**	*gg* *(D7)*	atgcttttgactactgg
tgtgcccggt	**acaacgg**	*cgac* *(D4)*	gcttttgactactgg
tgtgcccggt	**acaacgg**	*tg* *(D4)*	atgcttttgactactgg

[1] Putative N and P nucleotides are in italic, nucleotides coming from V or D are bold and underlined, while nucleotides matching a DH sequence (mentioned in brackets) are in bold. The IMGT gene tables and nomenclature were used (ImMunoGeneTics database, www.imgt.org). Additional information was found in rainbow trout genomic data (BAC AY872256 and https://www.genoscope.cns.fr/trout/); new D segments were named by the accession number of the scaffold/BAC followed by the location of the gene segment.
[2] Substitutions compared to the first sequence taken as a reference) are indicated in red.

CARYDDNAFDYW

VH5	DH or N/P diversity	JH5
tgtgcccggtaca	DH4: ataacaacggggg<br>DH5: ccatatagcggggt<br>DH7: cagaataacggc<br>DH8: tacactgggagc	acaatgcttttgactactgg
tgcgcccggta	**cg** *acg (D4/7)*	acaatgcttttgactactgg

CARYGGNAFDYW

VH5	DH or N/P diversity	JH5
tgtgcccggtaca	DH4: ataacaacggggg<br>DH5: ccatatagcggggt<br>DH7: cagaataacggc<br>DH8: tacactgggagc	acaatgcttttgactactgg
tgtgcccggtac	**acggggg** (D4)	caatgcttttgactactgg
tgtgcccggt	**acggggg** *t*(D4)	aatgccttttgactactgg
tgtgcccggt	**acggggg** *t*(D4)	aatgcttttgactactgg
tgcgcccggt	**acggggg** (D4)	caatgcttttgactactgg
tgtgcccggt	**acggggg** (D4)	caacgcttttgactactgg
tgtgcccggta	*t* **ggggg** (D4)	caatgcttttgactactgg
tgtgcccggt	**acggc** *ggt*(D7)	aatgccttttgactactgg
tgtgcccggt	**acggggg** a(D4)	aatgccttttgactactgg
tgtgcccgat	**acggggg** (D4)	caatgcttttgactactgg
tgtgcccggt	**acgg** *tggg*(D4/7)	aatgccttttgactactgg
tgtgcccggt	**acggc** *ggg*(D7)	aatgccttttgactactgg
tgtgcccggt	**acggc** *gg*(D7)	caatgccttttgactactgg
tgtgcccggt	**acggggg** *g*(D4)	aatgcttttgactactgg
tgtgcccggt	**acggggg** (D4)	caatgccttttgactactgg

CARYDGNAFDYW

VH5	DH or N/P diversity	JH5
tgtgcccggtaca	DH4: ataacaacggggg<br>DH5: ccatatagcggggt<br>DH7: cagaataacggc<br>DH8: tacactgggagc	acaatgcttttgactactgg

tgtgcccggtac	*g* **acgg (D4/7)**	caatgcttttgactactgg
tgtgcccggtac	*g* **acggg (D4)**	aatgcttttgactactgg
tgcgcccggtac	*g* **acgg (D4/7)**	caatgcttttgactactgg
tgtgcccggtac	*g* **acggg (D4)**	aatgcctttgactactgg
tgtgcccggta	*tg* **acggg (D4)**	aatgcttttgactactgg
tgtgcccggtac	*gat* **ggg (D4/7/8)**	aatgcctttgactactgg
tgcgcccggta	*tg* **acggg (D4)**	aatgcttttgactactgg
tgtgcccggta	*tg* **acgg (D4/7)**	caatgcttttgactactgg
tgtgccaggtac	*g* **acgg (D4/7)**	caatgcttttgactactgg
tgtgcccggtac	*g* **acgg (D4/7)**	caatgcctttgactactgg
tgtgcccggtac	*g* **acgg** *t* **(D4/7)**	aatgcttttgactactgg
tgtgcccggtac	*g* **acgg** *a* **(D4/7)**	aatgcttttgactactgg

CARYDNNAFDYW

VH5		JH5
tgtgcccggtaca	DH or N/P diversity<br>DH4: ataacaacggggg<br>DH5: ccatatagcggggt<br>DH7: cagaataacggc<br>DH8: tacactgggagc	acaatgcttttgactactgg

tgtgcccggt	**acg** *aca* (D4/7)	acaatgcttttgactactgg
tgtgcccggt	**acg** *ata* (D4/7)	acaatgcttttgactactgg
tgtgcccggt	**acg** *ata* (D4/7)	acaatgcctttgactactgg
tgtgcccggtac	*g* **ataa** t (D7)	aatgcttttgactactgg
tgtgcccggt	**acg** *ata* (D4/7)	acaatgctttcgactactgg
tgcgcccggt	**acg** *ata* (D4/7)	acaatgcttttgactactgg
tgtgcccggta	*tgata* (D?)	acaatgcttttgactactgg
tgtgcccggt	**acg** *aca* (D4/7)	acaatgcctttgactactgg
tgtgctcggt	**acg** *ata* (D4/7)	acaatgcttttgactactgg
tgtgcccggt	**acg** *aca* (D4/7)	acaacgcttttgactactgg
tgtgcccggt	**acg** *ata* (D4/7)	acaacgcttttgactactgg
tgtgcccggt	**acg** *ata* (D4/7)	acaatgcttttgactattgg
tgtgcccggta	*tgata* (D?)	acaatgcctttgactattgg
tgtgcccggta	*tgata* (D?)	acaatgcctttgactactgg

CARYNNNAFDYW

tgtgcccggtaca	DH or N/P diversity<br>DH4: ataacaacggggg<br>DH5: ccatatagcggggt<br>DH7: cagaataacggc<br>DH8: tacactgggagc	acaatgcttttgactactgg
tgtgcccggtaca	DH or N/P diversity	acaatgcttttgactactgg
tgtgcccggt	**acaa** *ta (D4)*	acaatgcttttgactactgg
tgtgcccggt	**ataa** *ta (D4/7)*	acaatgcttttgactactgg

tgtgcccggt ataacaa *(D4/7)* caatgcttttgactactgg
tgtgcccggt **ataa** *taat* *(D4/7)* aatgcttttgactactgg
tgtgcccggt **acaa** *ca* *(D4)* acaatgcttttgactactgg
tgtgcccggt **acaa** *ta* *(D4)* acaatgcctttgactactgg
tgtgcccggt **acaa** *ta* *(D4)* acaacgcttttgactactgg
tgtgcccggt **acaa** *ca* *(D4)* acaatgctttcgactactgg
tgtgcccggt **acaa** *ca* *(D4)* acaatgcctttgactactgg

CARYSGDAFDYW

tgtgcccggtaca DH or N/P diversity acaatgcttttgactactgg
DH4:ataacaacgggggg
DH5:ccatatagcggggt
DH7: cagaataacggc
DH8: tacactgggagc
DH14070_6526:
gccattcccgta

tgtgcccggtaca DH or N/P diversity acaatgcttttgactactgg
tgtgcccggtac *t* **ctggg** *g* (D8) atgcttttgactactgg
tgtgcccggtac *t* **ctgg** *ag* (D8) atgcttttgactactgg
tgtgcccggtac *tc* **cgggg** (D4/5) atgcttttgactactgg
tgtgcccggtac *t* **ctgg** *cg* (D8) atgcttttgactactgg
tgtgcccggtac *tc* **cggc** *g* (D7) atgcttttgactactgg
tgtgcccggtaca *gt* **ggag** (D8) atgcttttgactactgg
tgtgcccggtac *t* **ctgg** *ag* (D8) atgctttcgactactgg
tgtgcccggtaca *gt* **ggc** *g* (D7) atgcttttgactactgg
tgtgcccggtac *tc* **cggc** *g* (D?) atgcttttgactactgg
tgtgcccggta *ttc* **gggag** (D8) acgcttttgactactgg
tgtgcccggta *t* **agcgggg** (D5) atgcttttgactactgg
tgtgcccgata *ttc* **gggag** (D8) atgcttttgactactgg
tgtgcccggta **tagcgg** *cg* *(D5)* atgcttttgactactgg
tgtgcccggtac *c* **agcgg** *cg* *(D5)* atgcttttgactactgg
tgtgcccggtac *c* **agcgg** *tg* *(D5)* atgcttttgactactgg
tgcgcccggta *t* **agtgg** *cg* *(D5)* atgcttttgactactgg
tgtgcccggta *t* **agtgg** *cg* *(D5)* acgcttttgactactgg
tgtgcccggta *tt* **cgggg** *g* *(D5)* atgcttttgactactgg
tgtgcccggta **tatagcgg** *ag* (D5) atgcttttgactactgg
tgtgcccggta **tatag** *tggcg* *(D5)* atgcttttgactactgg
tgtgcccggta *t* ***attccgg*** *ag* *(D14070-6526)* atgcttttgactactgg
tgtgcccggta *t* ***attcggg*** **ag** *(D14070-6526)* atgcttttgactactgg

---

CARYDNNAFDYW

tgtgcccggtaca DH or N/P diversity acaatgcttttgactactgg
DH4: ataacaacgggggg
DH5: ccatatagcggggt
DH7: cagaataacggc
DH8: tacactgggagc

DH14070_6952:
acccgctatatga

tgtgcccggtaca	DH or N/P diversity	acaatgcttttgactactgg
tgtgcccggt	**acg** *aca* (D4/7)	acaatgcttttgactactgg
tgtgcccggt	**acg** *ata* (D4/7)	acaatgcttttgactactgg
tgtgcccggt	**acg** *ata* (D4/7)	acaatgcctttgactactgg
tgtgcccggt	*acg* **ataa** t (D7)	aatgcttttgactactgg
tgtgcccggt	**acg** *ata* (D4/7)	acaatgctttcgactactgg
tgcgcccggt	**acg** *ata* (D4/7)	acaatgcttttgactactgg
tgtgcccggt	*atgata* (D?)	acaatgcttttgactactgg
tgtgcccggt	**acg** *aca* (D4/7)	acaatgcctttgactactgg
tgtgctcggt	**acg** *ata* (D4/7)	acaatgcttttgactactgg
tgtgcccggt	**acg** *aca* (D4/7)	acaacgcttttgactactgg
tgtgcccggt	**acg** *ata* (D4/7)	acaacgcttttgactactgg
tgtgcccggt	**acg** *ata* (D4/7)	acaatgcttttgactattgg
tgtgcccggt	**tatga** *ta* (D14070_6952)	acaatgcctttgactattgg
tgtgcccggt	**tatga** *ta* (D14070_6952)	acaatgcctttgactactgg
