# Supplementary material for: Origin of Public Memory B Cell Clones in Fish After Antiviral Vaccination
Source: Front Immunol. 2018 Sep 27;9:2115. doi: 10.3389/fimmu.2018.02115 (PMC6170628; doi:10.3389/fimmu.2018.02115)
Supplement: Supplementary file 5 [file Data_Sheet_1.docx]

**Supplementary methods_._**

# Experimental design: fish, immunization protocols and sampling

Rainbow trout (homozygous gynogenetic clone B57, all females (Quillet et al., 2007)) were raised in the fish facilities of Institut National de la Recherche Agronomique (INRA, Jouy en Josas, France). One-year-old fish were placed in individual aquaria kept at 16ºC. Independent protocols of vaccination and viral boost were performed using the attenuated 25-111 variant of strain 07-71 of VHSV (Boudinot et al., 2001). The control group was sampled at the beginning of the protocol. The two other groups ("Vaccinated" and "Boosted"), received a first intramuscular (i.m.) injection of the virus (4x10^3^ PFU/fish) in a total volume of 50 µL. After five months, fish from "Vaccinated" group were sampled and fish from "Boosted" group were re-infected with the virus (i.m. injection of 1x10^6^ PFU/fish). Finally, samples from the boosted group were collected 1 month later (Figure 1). Trout were sacrificed by overexposure to 2-phenoxyethanol diluted 1/1000. Blood was extracted and let to clot at 4ºC overnight for serum extraction. The head kidney was remove to evaluate specific IgM secreting cells (ELISPOT, see below) and, the spleen was removed, fixed in RNA later (SIGMA, Aldrich) and kept at - 20ºC. Serum extraction was performed by centrifugation at 200 x g for 10 min, supernatants were collected and centrifuged at 1000 x g for 20 min. Serum was frozen at -20ºC to use in titration assays.

# ELISPOT

Briefly, leukocytes from head kidney were isolated via Histopaque 1077 (Sigma-Aldrich) density centrifugation. After washing the cells, samples containing 5x10^6^, 2,5x10^6^, and 1x10^6^ leukocytes were suspended in 1,5 mL of complete culture medium (GMEM + 10%FCS + Glu + Antibiotics) and plated on wells containing VHSV coated Nitrocellulose membrane. The coating was previously performed with 200ul of purified VHSV at 75ug/mL in PBS, an overnight at 4ºC. The remaining sites were blocked with 5% milk in PBS for 1 h at room temperature. VHSV specific IgM was then detected followed by a 1-h incubation in blocking solution in the presence of primary monoclonal antibody anti trout IgM (1.14, 1ug/mL). After few washes with PBS, the membranes were incubated by 1-h with secondary Ab goat anti-mouse IgG-Biotine (Amersham) and finally with Avidin-HRP. Finally, HRP activity was revealed by incubation at RT with 3-amino-9-ethyl-carbazole (AEC system) and allowing to develop until distinct spots formed. The reaction was stopped by washing the wells under running water and the plates were allowed to air dry before being counted.

# CDR3 length spectratyping analysis

A first amplification (PCR1) using a forward primer specific for a family or a subset of VH segments in combination with a reverse primer specific for Cµ, Cτ sequences (Table S1) was performed as follows: 1 µl cDNA was used as template for PCR1 using 0.4 mM each dNTP, 0.4 µM each primer (forward: VH_family_ _specific_, reverse: C_isotype_ _specific_), and 0.025 u µl^-1^ of GoTaq DNA polymerase (Promega) in 1x reaction buffer with 2 mM MgCl_2_ (95°C for 5 min; 40 cycles of 95°C 45 s, 60°C 45 s, 70°C 45 s; 70°C 10 min). In a second step, VH-C PCR (2 µl) products were subjected to run off reactions (PCR2) using 5’ 6-FAM- fluorescent C internal, isotype specific, reverse primers (using 0.4 mM each dNTP, 10 pmol fluorescent reverse primer, and 0.025 u µl^-1^ of GoTaq DNA polymerase (Promega) in 1x reaction buffer with 2 mM MgCl_2_ (95°C for 5 min; 5 cycles of 95°C 1 min, 60°C 1 min, 70°C 2 min; 70°C 10 min)). Two µl of run-off product were analyzed using an ABI 7900HT sequencer (Applied BioSystems). CDR3 length distributions were analyzed using GeneMapper (Applied BioSystems) and ISEApeaks software (Collette and Ferrandiz, 2004; Collette and Six, 2002) to extract and analyze spectratype data for each VH-C combinations.

# Primers used for CDR3 spectratyping analysis.

| Name | Sequence | Expression | | a | Amplified VH segment b |
| --- | --- | --- | --- | --- | --- |
|  |  | ***IgM*** | ***IgT*** | |  |
| VH1.1-F | AGCAGTGATGGTGGCAGCACT | +/- | +/- | | IGHV1S1; Sc2184(31913c)c |
| VH1.2-F | CAATTAGTGATTCAAGCAGTTATA | - | - | | IGHV1S2 |
| VH1.3-F | CAGCGCATTATGACATTAGAAATA | - | - | | IGHV1S3;Sc68806(2195c) |
| VH1.4-F | TTCTGCACCAAGTGGAGCTGACAA | - | - | | IGHV1S5 |
| VH1.5-F | TATGATAGTGCTGAAATCTACTAC | +/- | +/- | | IGHV1S4; IGHV1S6; IGHV1S7 ; Sc5246(2097) |
| VH2-F | GGTCTGAGAGCAGAGGACTCTGC | - | - | | IGHV2S1 to IGHV2S3 ; Sc24807(2063) , |
|  |  |  |  | | Sc2184(62223c), Sc4731(39130), |
|  |  |  |  | | Sc4731(40316), Sc2931(46301), |
|  |  |  |  | | Sc30311(2218), Sc71265(956), |
|  |  |  |  | | Sc79393(814) |
| VH3.1-F | CATGTGTGGCAGTGGTAACATA | + | + | | IGHV3S1 to IGHV3S4; Sc5246(8805) |
| VH3.2-F | TTGTTACAATGGTAACACATATG | - | - | | Sc4000(23955) |

| VH4.1-F | ACTCTGGTTCAACAGATGCTCCAGTC | + | + | IGHV4S1 |
| --- | --- | --- | --- | --- |
| VH4.2-F | TGGATTGGAAGAATGAACACTGG | +/- | - | Sc33539(850) |
| VH5.1-F | TACACACTGGTGGATCGAGTC | + | + | IGHV5S2; IGHV5S5; IGHV5S6; IGHV5S7; IGHV5S8; IGHV5S9; Sc5246(35108) |
| VH5.2-F | CAGCACAGCTAGTACACCCAT | + | + | IGHV5S3; Sc20487(6839) |
| VH5.3-F | TGGATTGC(C/T)TATAGTTATAGTAC | + | +/- | IGHV5S4; Sc2184(10321c); Sc2931(29345); Sc54572(565) |
| VH5.4-F | ATATTAGCACACAGAGTAATCC | + | +/- | IGHV5S1 |
| VH6-F | CTGAAAAA(C/T)AAGTTCAGCCTC | + | -/+ | IGHV6S1 to IGHV6S9 ; Sc4731(22733); Sc7987(16784c); Sc934(366199c);  Sc11506(7595); Sc4731(18064); Sc4731(1176) |
| VH7.1-F | GTTCAGCATTTCAACACATGC | -/+ | -/+ | IGHV7S1 |
| VH7.2-F | CAAGTTCAGCTTTAGAAGAGAC | +/- | -/+ | Sc4731(5937) |
| VH8-F | AAGGACAGCA(G/C)(A/T)AATTTCTATCTG | + | + | IGHV8S1 to IGHV8S11 ; Sc9908(8999) |
| VH9.1-F | CTGGAACCACTGCTTATTATGC | + | - | IGHV9S3 ; IGHV9S4; IGHV9S5; IGHV9S6; Sc2931(8571c) |
| VH9.2-F | TGGCACTGGCACTGTATTTGC | + | + | IGHV9S1; IGHV9S2; Sc37982VH1 ;  Sc15217(5572); Sc2184(39160c); Sc2931(6853) |
| VH10-F | AGCAAGATCACTTCTAGATATG | -/+ | - | IGHV10S1 |
| VH11-F | TGGAGTGGATTGGGATCATCTG | -/+ | + | IGHV11S1; Sc4731(42369); Sc7987(19414c) |
| VH12-F | GGACCATCTATTATGATGGAAGC | + | - | IGHV12S1 |
| VH13-F | GATACCTGTGTAGTAGTAGTAGC | +/- | -/+ | IGHV13S1; Sc2184(55767c) |
| Cµ2-R | AGAGACGGCTGCTGCAGATATTCC |  |  |  |
| Cµ1-R* (internal)d | CACATTGCGCAAGAGGGAACAA |  |  |  |
| Cτ2-R | GATGTCGTTAGAAGGGGTTCCA |  |  |  |
| Cτ1-R* (internal)d | GTTCCACAGTTCATAAGAGTG |  |  |  |

a Expression of VHs in splenic IgM and IgT rearrangements from control trouts. (+) detected in all samples; (+/-) in at least 50%; (-/+) less

than 50% and (-) no detected.

b VH segments amplified, including those already present in IMGT database and those identified on genome scaffolds.

c Scaffold number, in brackets the position of first VH nucleotide and “c” means complementary.

d The 5´6-FAM fluorescent version was used for the run off reaction.

# Length of the run-off products (max and min values for each profile).

| IgM |  |  | IgT |  | |
| --- | --- | --- | --- | --- | --- |
| VH primer | Start | End | VH primer | Start | End |
| 1.1 | 226 | 259 | 3.1 | 229 | 274 |
| 3.1 | 232 | 265 | 4.1 | 245 | 290 |
| 4.1 | 234 | 267 | 5.1 | 228 | 273 |
| 4.2 | 248 | 280 | 5.4 | 237 | 282 |
| 5.1 | 231 | 264 | 8.1 | 169 | 214 |
| 5.2 | 227 | 260 | 9.2 | 220 | 265 |
| 5.4 | 234 | 266 |  | | |
| 6.1 | 189 | 222 |  |  |  |
| 8.1 | 189 | 222 |  |  |  |
| 9.1 | 189 | 222 |  |  |  |
| 9.2 | 189 | 222 |  |  |  |
| 12 | 230 | 263 |  |  |  |

**Implementation of a barcoded IgH cDNA sequencing analysis of the clonal complexity of trout Ab repertoire**

Preparation of Illumina MiSeq libraries

Libraries for Illumina deep sequencing were prepared as described by Vollmers et al (Vollmers et al., 2013). For cDNA barcoding, the primers used for second strand cDNA contained 15 random nt (Figure S1 and Table S1). The location of the first Cµ primer in the Cµ2 domain avoids coamplification with IgHδ mRNA, which contains a Cµ1 domain. The following VH/C combinations were analyzed: VH4-Cµ (primer VH4.1), VH5-Cµ (primer VH5.1), VH8-Cµ (primer VH8.1), VH4-Cτ (primerVH4.1), VH5-Cτ (primer VH5.4) and VH9- Cτ (primer VH9.2). Final PCR with Illumina adapters were purified using Agencourt AMPureXP beads (Beckman Coulter, Brea, CA) in a 1 to 0.95 DNA per bead ratio. Libraries quality was assessed on a "DNA High

Sensitivity" chip " with a bioanalyzer instrument (Agilent Technologies, Santa Clara, CA). Equal amounts of libraries were pooled for multiplexing, and pools were sequenced in paired-end 2x300pb runs using a MiSeq instrument (Illumina) and the MiSeq Reagent Kit v3 (600 cycles) (Illumina) according to the manufacturer recommendations. Twenty percent of an Illumina PhiX library to compensate for sequence low diversity. Fastq files were treated with Cutadapt 1.3 to remove sequencing adaptors. Read quality was assessed with Fastqc 0.10.1.

Development of a barcoded IgH cDNA sequencing strategy

Using Illumina Miseq technology, we developed a consensus read sequencing approach based on the incorporation of a unique random barcode in each cDNA molecule produced at the reverse transcription step as described for human IgH in (Vollmers et al., 2013). Figure S1 summarizes the approach we used for the rainbow trout IgH repertoire. Random labels (UID) permit a more accurate quantification of clonotype frequencies, and a better correction and evaluation of the error rate due to PCR and sequencing. Individuals were indentified by Fish Barcodes (FBD) Barcoded cDNA libraries were produced from the same RNA samples used for CDR3 length spectratyping, and sequenced. Based on sequence barcoding, clonotype frequencies were analyzed as described below.

# Primers used to prepare amplicon libraries.

| ***cDNA*** |  |
| --- | --- |
| Cµ2-R | AGAGACGGCTGCTGCAGATATTCC |
| Cτ2-R | GATGTCGTTAGAAGGGGTTCCA |
| ***ds cDNA*** |  |
| Rd2p_UID_VH4.1 | GTGACTGGAGTTCAGACGTGTGCTCTTCCGATCT(N)15ACTCTGGTTCAACAGATGCTCCAGTC |
| Rd2p_UID_VH5.1 | GTGACTGGAGTTCAGACGTGTGCTCTTCCGATCT(N)15TACACACTGGTGGATCGAGTC |
| Rd2p_UID_VH5.4 | GTGACTGGAGTTCAGACGTGTGCTCTTCCGATCT(N)15ATATTAGCACACAGAGTAATCC |
| Rd2p_UID_VH8.1 | GTGACTGGAGTTCAGACGTGTGCTCTTCCGATCT(N)15AAGGACAGCA(G/C)(A/T)AATTTCTATCTG |
| Rd2p_UID_VH9.2 | GTGACTGGAGTTCAGACGTGTGCTCTTCCGATCT(N)15TGGCACTGGCACTGTATTTGC |
| ***PCR*** |  |
| Rd1_6N_Cmu1 | AATGATACGGCGACCACCGAGATCTACACTCTTTCCCTACACGACGCTCTTCCGATCTCACATTGCGC AAGAGGGAACAA |
| Rd1_4N_Cmu1 | AATGATACGGCGACCACCGAGATCTACACTCTTTCCCTACACGACGCTCTTCCGATCTCATTGCGCAA  GAGGGAACAAAG |
| Rd1_2N_Cmu1 | AATGATACGGCGACCACCGAGATCTACACTCTTTCCCTACACGACGCTCTTCCGATCTTTGCGCAAG AGGGAACAAAGTC |
| Rd1_6N_Ctau1 | AATGATACGGCGACCACCGAGATCTACACTCTTTCCCTACACGACGCTCTTCCGATCTTTCCACAGTT CATAAGAGTGA |
| Rd1_4N_Ctau1 | AATGATACGGCGACCACCGAGATCTACACTCTTTCCCTACACGACGCTCTTCCGATCTCCACAGTTCA  TAAGAGTGAGT |
| Rd1_2N_Ctau1 | AATGATACGGCGACCACCGAGATCTACACTCTTTCCCTACACGACGCTCTTCCGATCTACAGTTCATA AGAGTGAGTAG |
| Rd2_FBD_1 | CAAGCAGAAGACGGCATACGAGATCGTGATGTGACTGGAGTTCAGACGTGTGCTCTTCCGATCT |
| Rd2_FBD_2 | CAAGCAGAAGACGGCATACGAGATACATCGGTGACTGGAGTTCAGACGTGTGCTCTTCCGATCT |
| Rd2_FBD_3 | CAAGCAGAAGACGGCATACGAGATGCCTAAGTGACTGGAGTTCAGACGTGTGCTCTTCCGATCT |

*Sequencing analysis: filtering, alignment, production of consensus from paired reads*

Fastq files from the Miseq were treated with Cutadapt 1.3 to remove sequencing adaptors, and read quality was assessed with Fastqc 0.10.1. Read pairs in which R1 or R2 length were <150bp or >350bp were discarded, as well as those in which exact V and C primer sequences were not present in R1 (respectively R2). R1 and R2 were defined as in the Illumina Indexed Sequencing guide <http://emea.support.illumina.com/content/dam/illumina-support/documents/documentation/system_documentation/miseq/indexed-> sequencing-overview-guide-15057455-04.pdf. VH primer sequence started at the position 16 of R1, since the first 15 positions constituted the cDNA barcode sequence. R1, and reverse complemented R2 sequences were aligned using Muscle (Edgar, 2004). Read pairs in which R1/R2 overlap contained more than 10 mismatches were discarded. For the others, a consensus sequence (ConS_R1/R2_) was computed, keeping R1 sequence up to the V proximal half of the alignment length, and R2 reverse complement sequence for the C proximal half of the alignment.

*Sequence annotation*

Each ConS_R1/R2_ was translated to the three *forward frames. To identify the location of the CDR3, each translation was scanned for the following motif:* **YYC*WGXG**, where ‘*’ means any character found *n* times with n ∈ {0 - 20}, and ‘X’ means any character found once. To identify a valid CDR3, the motif had to be found within a given region of the ConS _R1/R2_ (after position 42 for VH4.1, 39 for VH5.1, 20 for VH8.1, 43 for VH5.4 and 39 for VH9.2). ConS _R1/R2_ in which this motif could not be found, including all sequences in which VDJ rearrangements led to frameshift, were discarded. The CDR3 was defined as previously between C_105_ and W_117_. Importantly, to allow easy comparison between fluorescence CDR3 length profiling and data from deep sequencing, ConS_R1/R2_ in which CDR3 contained a STOP in frame were not discarded at this stage of the

analysis. To filter potential artifacts, sequences in which the CDR3 was longer than 22AA for Cµ sequences and longer than 35AA for Cτ were discarded. J segments were annotated according to IMGT gene tables; one mismatch was allowed in the J (nucleotide) sequences to name the segment. For each ConS, we therefore produced an annotation comprising: a barcode, a VH subgroup defined by the V primer used for the amplification (see Table S1 for the detailed specificity of each VH primer), a C type (µ or τ; the primers used matched known C sequenced from rainbow trout IgHA and B loci, therefore co-amplified them all), an in-frame CDR3 sequence, and a J segment. It is important to note that in the current state of annotation of the rainbow trout Ig gene repertoire and polymorphism, our V and C annotation defines V family subsets and isotypes, respectively, rather than unique genes. Since we used an isogenic clone of rainbow trout in which every locus is in homozygous configuration, the complexity of annotation was limited to a single haplotype.

*Definition of a unique cDNA molecular barcoding to determine clonotype count*

Amplification bias is a major issue for producing accurate descriptions of immune repertoires using deep sequencing. To solve this problem, different systems of cDNA barcoding have been developed with random UIDs incorporated on one or both side(s) of the templates during reverse transcription. We offset PCR biases by developing a unique molecular identifier (MID) based on the combination of (1) the random barcode incorporated in the primer used for the second strand cDNA synthesis (see above) and (2) the CDR3 sequence.

Sequencing error correction based on the incorporation of the random tag was performed as follows: for each set (seqS) of R1/R2 consensus paired sequences from a given sample, sharing the same VH, JH, C and UID, the count of each particular sequence was computed. The most frequent sequence was selected; all (nt) sequences with only one mismatch (nt difference, not deletion or insertion) were aggregated to it, *ie* were attributed its nt sequence, and a MID defined as above, based on the CDR3 sequence and UID. In the sequel, the most frequent sequence was subjected to the same protocol, and this iterative process pursued until all sequences from the seqS were attributed a MID. Each clonotype being defined by: a V tag, a C tag, a J annotation, and a CDR3 sequence, its expression level was computed by counting the corresponding MID barcodes, as defined above.

This process allowed to cluster sequences of which ConS_R1/R2_ differed at one position; with a error rate of about 10^-3^per nucleotide (see below "Estimating the error rate in Illumina sequence datasets"), this method led to an efficient error correction. In the (rare) cases where ConS_R1/R2_ from a seqS differed at more than one position, even if they shared the same UID, sequences were considered distinct, and were conservatively attributed different MID.

Validation of the sequencing approach

To validate our approach, CDR3 length distribution was computed for each VH/C combination and compared to the PCR-based profiling described above. As shown in figure S7, these “virtual spectratypes” and the fluorescence profiles were very similar, showing that our sequencing and analysis protocols did not distort the distribution of junction lengths. We also checked the reproducibility of our sequencing approach by analyzing independent libraries prepared in parallel from aliquots of the same RNA and sequenced in different Mi-Seq runs. For example, 7991 VH5Cµ clonotypes of a total of 41833 were shared by the two replicates Rep1 and Rep2 (Figure S7). When only abundant clonotypes were considered (count number >=5 or 10), most clonotypes were present in both Rep1 and Rep2, indicating a robust detection of abundant clonotypes. Additionally, shared clonotypes were found at similar abundance (Figure S7) (R^2^=0.92 for VH5Cµ; R^2^=0.80 for VH4Cτ) in R1 and R2. As demonstrated previously for the same strategy (Vollmers et al., 2013), the lesser sharing of low abundance sequence is due to incomplete coverage. Thus, although the sequencing depth is too low to describe the whole clonotype diversity present in the samples, our data provide an accurate account of the population of large clonotypes.

We also estimated the error rate via two independent methods, either from the 50 unique clonotype ID (named "MID", see Material and Methods, section "Definition of a unique cDNA molecular barcoding to determine clonotype count") having the highest number of reads or from 500 MIDs having two read pairs drawn at random (see Material and methods); in average, the error rate estimated by both methods over all VH-C combinations analyzed was about 2.10^-3^ per base pair (see Material and methods about the error rate in different regions of the VH/C PCR products).

To get insight about the response to vaccination or boost (see corresponding sections below), we followed three approaches: (1) we analyzed the global characteristics of Ab repertoires in control (group "Ctl"), vaccinated (group "Vac") and boosted fish (group "Bst"); (2) we then focused on the most frequent clonotypes in each V-C combination, among which responding clonotypes are likely to be found (3) finally, we focused on clonotypes shared by 3 fish or more per groups (noted "HS", for highly shared), targeting the public response (*i.e.*, by definition, found in all fish within an immunized group).

*Estimating the error rate in Illumina sequence datasets*

Two approaches were followed to assess the errors introduced in Ig sequences:

1. we first analyzed the differences between R1 and R2 sequences. For a given sample, we randomly selected 10.000 pairs of reads {R1; R2} ; reads of each pair were aligned using muscle, and the R1/R2 overlap region was considered for further steps (in most cases, R1 and R2 reverse complement fully overlapped from V to C primers). The alignment was divided in three regions: before CDR3 (V side), CDR3 and after CDR3 (C side). For each region, the proportion of positions in which R1 and R2 were different was computed. This

analysis across all reads used in this study produced the following results:

Before CDR3 : 72616 / 84373317 = 0.000861; CDR3 : 18925 / 24106448 = 0.000785

After CDR3 : 258520 / 41251357 = 0.00627.

This assessment is consistent with the typical error distribution of the Illumina protocol used in this work; R1 and R2 quality do not vary in the same way, R1 quality getting degraded quicker than R2. Considering the location of CDR3, we therefore determine a R1/R2 "consensus" for each pair as described above.

1. to assess the error rate in our processed data, we used primer-based random barcoding to compare sequences amplified from a given cDNA molecule, and to calculate the corresponding error frequency. For each V/C combination dataset, and for each individual, the 50 most represented MIDs were considered, and a R1/R2 consensus (ConS_R1/R2_) computed for each pair of reads having these "topMID".

To determine the *substitution* rate, only ConS_R1/R2_ of the most frequent length were considered for each MID, to build a "MID-consensus" by considering the most frequent nucleotide at each position. The substitution rate was estimated by the frequency of mismatch outside CDR3, between all ConS_R1/R2_ and their MID consensus sequence. The same procedure was followed for a sampling of 500 MIDs randomly chosen between those represented by at least three {R1; R2} pairs of sequences.

To estimate the indel rate, we counted all size shifts of ConS_R1/R2_, compared to the corresponding MID- consensus. In this way, indels could be detected only outside CDR3, since sequences with out-of-frame CDR3 had been discarded from the dataset in the beginning of the treatment. Counts were then normalized over the length of sequences outside CDR3, for each V/C combinations. These analyses led to the following results: Global mismatch rate per nuc (part=Top50) 25003/11190510 = 2.2 10^-3^

Global mismatch rate per nuc (part=Random500) 59124/30643411= 1.9 10^-3^ Global indel rate (part=Top50) 11959/12127034= 9.8 10^-4^

Global indel rate (part=random500) 28642/32805544= 8.7 10^-4^

With an error rate of ≈10^-3^, we are therefore confident that our computation of CDR3 consensus and counts, hence our observations on the variations of clonotype distributions, are reliable.

*Dataset normalization by subsampling and analysis of sets of large clonotypes*

When we seek to quantify the number of fish containing a given clonotype, such a clonotype is much more likely to be found in fish whose sequencing yielded large numbers of sequences, although this is not necessarily related to the biological features of their repertoires. Hence, using frequencies is not an option in that case. To bring all fish on an equal footing while preserving the clonotypes relative frequencies, we resort to subsampling. This is an *in silico* equivalent of the sampling process occurring during sequencing, except that the number of sequences obtained in the end is controlled and equal in all fish.

Individual fish datasets, for each VH/C combination and in all three analyzed conditions, were therefore normalized by random subsampling without replacement. Ten subsamples of 7000 MID were performed (see total numbers of MID for each point in Table S7), and the average results were computed and analyzed. The relatively low number of MID subsampled was imposed by the smallest individual MID count (n=7917 for VH4- Cτ); the conclusions of the analyses are supported by the highly consistent results produced by distinct subsamples.

To focus on large clonotypes for each VH/C combinations, we considered the 50 most frequent clonotypes detected in each fish of a group. We chose this threshold (*i.e.* 50) to capture all clonotypes expressed more than once in average, in normalized (subsampled) individual datasets. We also verified that all conclusions drawn from this analysis were supported by lists contained the 100 or 150 most frequent clonotypes. Aggregating these individual lists from all fish of a given group (*i.e.* controls, vaccinated or boosted) produced non redundant sets of Top clonotypes, noted TCS_Ctl_, TCS_Vac_ or TCS_Bst_, of which intersections are presented in Venn diagrams shown in Figure 3 and S4. Note that since TCS are determined from the full datasets (*i.e.* not from subsampling), there is no error bars or standard deviation associated to the Venn diagrams.

*Definition of CDR3 "similarity" classes*

We defined sets of sequences in which the CDR3 region differed by two conservative AA substitutions at most, to investigate the fraction of clonotypes somewhat similar to the TOP50 clonotypes and to those involved in the response to the virus. Amino acid substitutions were considered conservative when exchanging residues with similar properties, *i.e.* belonging to the same classical category. We considered four categories:

**Aliphatic** : Isoleucine (I) Leucine (L) Valine (V)

**Aromatic** : Phenylalanine (F) Tryptophan (W) Tyrosine (Y) Histidine (H)

**Charged and acidic**: Glutamine (Q) Asparagine (N) Glutamic acid (E) Lysine (K) Arginine (R) Aspartic acid (D) Histidine (H)

**Tiny** : Alanine (A) Glycine (G) Serine (S) Cysteine (C)

Similarity classes did not aim at defining true lineages of cell with receptors produced by somatic hypermutation from an original ancestor. Instead, CDR3 similarity classes constitute a good proxy to model the variety of CDR3 sequences typically implicated in public responses. For a given condition (Controls, vaccinated or Boosted) pairs of clonotypes were formed from any of the 6 distinct sets of two individual repertoires among the four individuals/condition; clonotypes of a pairs were considered "similar" when the AA sequence of their CDR3 differed at most by two conservative substitutions as described above.

The numbers of pairs of "similar" clonotypes were counted for each repertoire comparison, and the average and

standard deviation represented in figure 6. These calculations were performed for different subsamplings, and produced quasi-identical results. When comparing similarity of VH/C clonotypes to the 8 VH5JH5 clonotypes, averages were taken over 4 individual repertoires for each conditionconsidered.

# Development of a computational model of IgH rearrangement (see also Fig S6)

We constructed a computational model of IgH VDJ rearrangements, which produces *synthetic generated repertoires*, *i.e.* the set of clonotypes realized from the rearrangement process alone. To train the model and determine its parameters, we used the non-productive (NP) sequences of our datasets (either out-of- frame or with an in-frame premature stop codon), whose composition exhibits the statistics of the rearrangements since they are not subjected to selection.

# Number of sequences used to train the computational model of IgH rearrangement

|  | VH4Cµ | VH5Cµ | VH8Cµ | VH4Cτ | VH5Cτ | VH9Cτ |
| --- | --- | --- | --- | --- | --- | --- |
| Number of NP sequences  (paired) | 116811 | 179811 | 846561 | 164106 | 279766 | 864016 |
| Proportion in the  dataset for each VH/C | 6.2% | 3.1% | 4% | 3.9% | 5.1% | 5.3% |

We employed an existing computational tool suite, IGoR (Marcou et al., 2017), that learns a generative probabilistic model of the IgH VDJ recombination process and generates corresponding synthetic receptor sequences. The parameters were inferred for each VH/C combination sequenced in this work, from the corresponding sets of non-productive sequences. The IMGT gene tables and nomenclature were used (ImMunoGeneTics database, www.imgt.org). Additional segments found in rainbow trout genomic data (BAC AY872256 and http[s://w](http://www.genoscope.cns.fr/trout/))ww[.g](http://www.genoscope.cns.fr/trout/))e[noscope.cns.fr/trout/)](http://www.genoscope.cns.fr/trout/)) were named by the accession number of the scaffold/BAC followed by the location of the gene segment.

Briefly, nucleotide sequences of non-productive transcripts directly reflect the statistics of the recombination process, convolved with noise from mutations and sequencing error. The recombination process is defined by the use of a given set of V, D, and J genes, and by the deletions and insertions occurring at the VD and DJ junctions. The parameters of this process are therefore the probabilities of each of these events. The model’s parameter values were inferred from measured non-productive sequences by maximizing the likelihood for these sequences to be generated. Reference (Marcou et al., 2017) provides a computationally-efficient implementation of the model and this maximization problem. It uses a variational expectation maximization algorithm, which increases the likelihood of the dataset by updating the event frequency parameter values to their empirical frequencies given the current values, *i.e.* to the average likelihood of the sequences in which they occur. The procedure is iterated until the likelihood converges to a maximum. The model adds point mutations to the result of the process at a specific error rate to account for the joint effect of mutations and sequencing errors. This error rate is inferred along with the event probabilities. The inferred model can then be used to generate synthetic sequences, with and without errors, along with their probability of generation.

Distributions of the latter were used to calculate the rearrangement diversity via their Shannon entropy discussed in Section 4.1. The inferred model was also used to obtain a simulated set of top abundant sequences with which to perform the sharing analysis and compare to the controls in Section 4.3. Specifically, we computed the generation probability of the measured top abundant clones of the control dataset using the inferred models. We then sampled sequences from the synthetic repertoire in such a way that their distribution of generation probabilities matched that of the measured top abundant clones.

# Identification of "public" clonotypes

To identify "public" or "highly shared" responses, we selected clonotypes that were (1) ***detected*** in at least three fish per group, either in Vaccinated or Boosted groups ("**shared clonotypes**") and (2) **overexpressed** more than *k* times in average between control and vaccinated (or boosted) fish. Since it is typically accepted that an activated B cell clone is amplified and typically expresses > 100 times more IgH transcripts than a naive cell, we took a conservative value *k*=50. To take into account milder changes, we also considered *k*=25 and *k*=10. In a third step, we examined whether the level of expression of these **overexpressed shared clonotypes** was consistently high across immunized fish of the vaccinated and boosted groups. Clonotypes being present in most individuals of immune groups, differentially expressed between controls and immunized fish, and **well- expressed across Vac and/or Bst groups**, were considered as part of the public response to the virus.

**References**

Boudinot, P., Boubekeur, S., and Benmansour, A. (2001). Rhabdovirus infection induces public and private T cell responses in teleost fish. Journal of immunology *167*, 6202-6209.

Collette, A., and Ferrandiz, M.E. (2004). A Profound Alteration of Blood TCRB Repertoire Allows Prediction of Cerebral Malaria 1. Journal of immunology (Baltimore, Md : 1950) *173*, 4568-4575.

Collette, a., and Six, a. (2002). ISEApeaks: an Excel platform for GeneScan and Immunoscope data retrieval,

management and analysis. Bioinformatics (Oxford, England) *18*, 329-330.

Edgar, R.C. (2004). MUSCLE: multiple sequence alignment with high accuracy and high throughput. Nucleic acids research *32*, 1792-1797.

Marcou, Q., Mora, T., and Walczak, A.M. (2017). IGoR: a tool for high-throughput immune repertoire analysis. arXiv *1705.08246*.

Quillet, E., Dorson, M., Le Guillou, S., Benmansour, A., and Boudinot, P. (2007). Wide range of susceptibility to rhabdoviruses in homozygous clones of rainbow trout. Fish & shellfish immunology *22*, 510-519.

Vollmers, C., Sit, R.V., Weinstein, J.A., Dekker, C.L., and Quake, S.R. (2013). Genetic measurement of memory B-cell recall using antibody repertoire sequencing. Proceedings of the National Academy of Sciences of the United States of America *110*, 13463-13468.
