# Supplementary material for: Origin of Public Memory B Cell Clones in Fish After Antiviral Vaccination
Source: Front Immunol. 2018 Sep 27;9:2115. doi: 10.3389/fimmu.2018.02115 (PMC6170628; doi:10.3389/fimmu.2018.02115)

Figure S1. Spectratyping set-up and construction of Illumina libraries

(A) Schematic representation of a rainbow trout Ig heavy chain locus (IgH), with typical IgHμ, δ and τ transcripts. V and C primers used for CDR3 length spectratyping and library amplification are represented. Note that IgHμ and δ comprise a Cμ1 domain just after the J segment. Hence, IgM specific cDNA synthesis was performed using a primer located in Cμ2, and the 15nt random sequence barcode had to be included in the primer used for the second cDNA strand synthesis, on the V side (see 1B). (B) Library preparation: IgH RNA molecules were reverse transcribed using primers specific for Cμ or Cτ. Second strand cDNA synthesis was performed using pools of VH primers located in the framework region 3 (FR3). These primers contained the 15 random nt (UID) and partial sequencing adapter sequences (Rd2p). cDNA was then PCR amplified, using primers with Illumina MiSeq sequencing adapters (Rd2p and Rd1) and with a Fish specific barcode (FBD) in the forward primer.

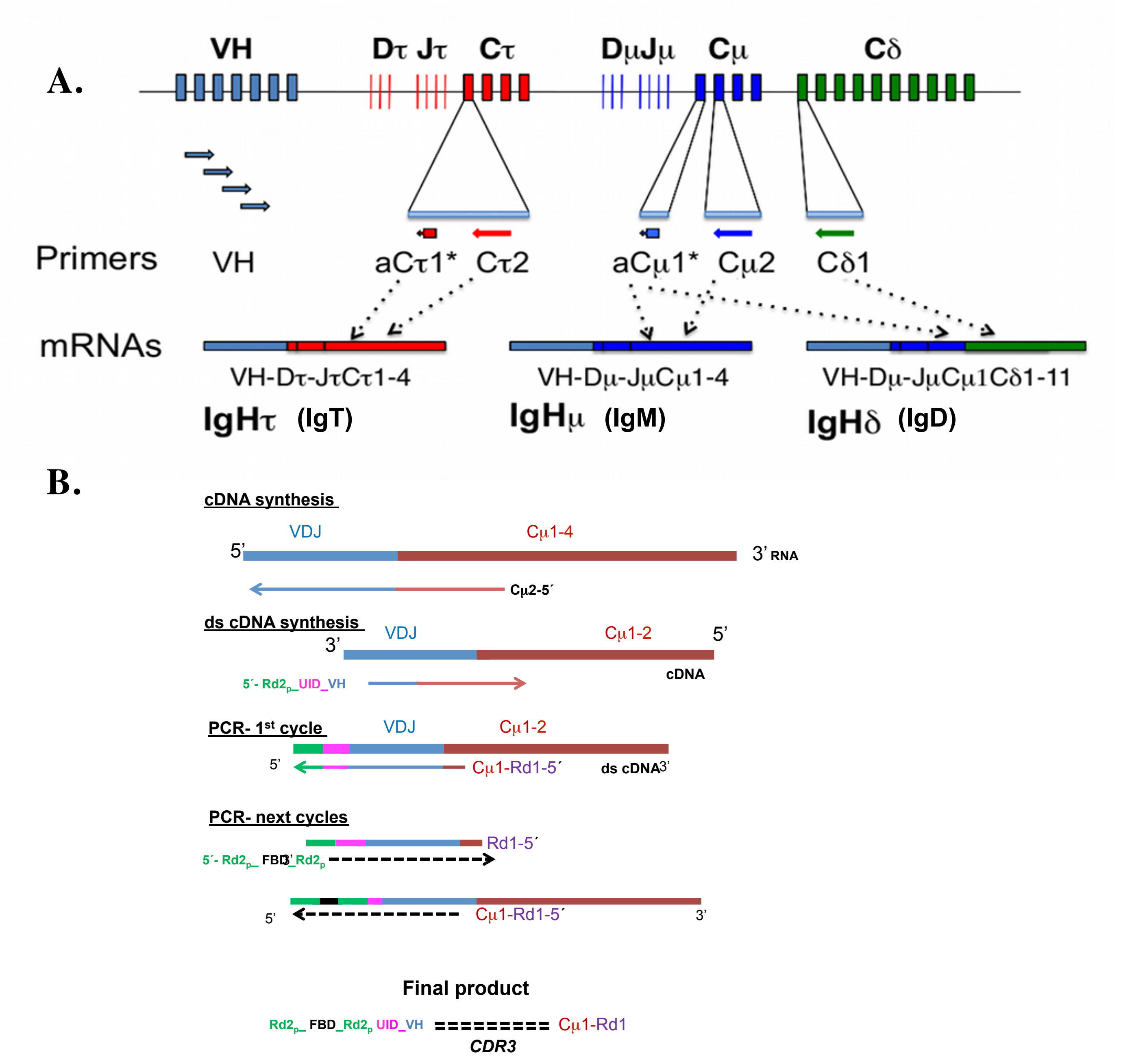

Supplement: Supplementary file 6 [file Image_1.pdf]
