# Supplementary material for: Origin of Public Memory B Cell Clones in Fish After Antiviral Vaccination
Source: Front Immunol. 2018 Sep 27;9:2115. doi: 10.3389/fimmu.2018.02115 (PMC6170628; doi:10.3389/fimmu.2018.02115)

Figure S2. Validation of the consensus read sequencing. A. Comparison of CDR3 length distributions from fluorescence spectratyping (in blue) or computed from deep sequencing data (in red). Profiles show IgM profiles from the fish Vac2 (group «Vaccinated»). Similar results were observed for IgT. The corresponding primer combinations are indicated. Y axis: fluorescence arbitrary units, X axis: length of the run-off products (max and min values for each profile are given in Table S1. B. Venn diagrams showing the overlap of clonotype repertoires detected by parallel sequencing (from different runs) of replicate libraries from the same RNA preparation. Numbers of sequences shared or unique to replicates are indicated for two VH/Ccombinations, for all clonotypes (MID≥1) or clonotypes found at least 5 (MID≥5) or 10 (MID≥10) times. C. For sequences shared between replicates, the abundances are shown as a scatterplot, for the same VHC combinations.

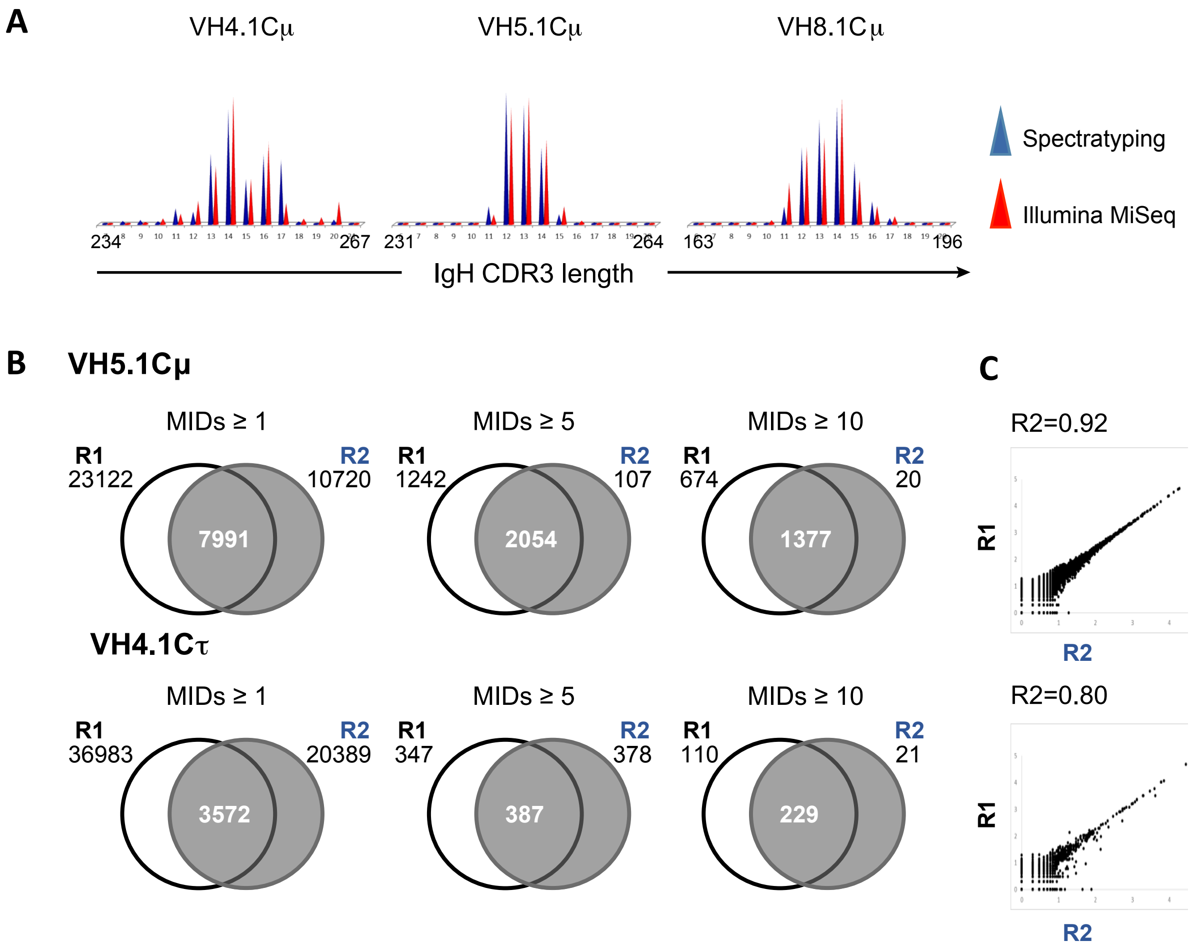

Supplement: Supplementary file 7 [file Image_2.pdf]
