# Supplementary material for: Origin of Public Memory B Cell Clones in Fish After Antiviral Vaccination
Source: Front Immunol. 2018 Sep 27;9:2115. doi: 10.3389/fimmu.2018.02115 (PMC6170628; doi:10.3389/fimmu.2018.02115)

Figure S3. Spectratypes observed in control, vaccinated, and boosted fish for multiple VH combined with Cμ or Cτ.

A. IgM profiles for the 12 expressed VH families. B. IgT profiles for the 6 expressed VH families. A bimodal distribution of CDR3 length was generally observed for some VH like VH4 and VH8; the bell-shaped extension of the distribution towards longer junctions was due to the presence of a particularly long D segment (ACTATACAGTTACAGTTTGGGCTT(T/C)TTATT(C/T)GAGAGC) in the τ locus. The long Ig junctions, which had been noticed in the original report describing this isotype (Hansen et al., 2005), expressed this segment in our dataset. The start and end position of each profile are given for each VH/C combination.

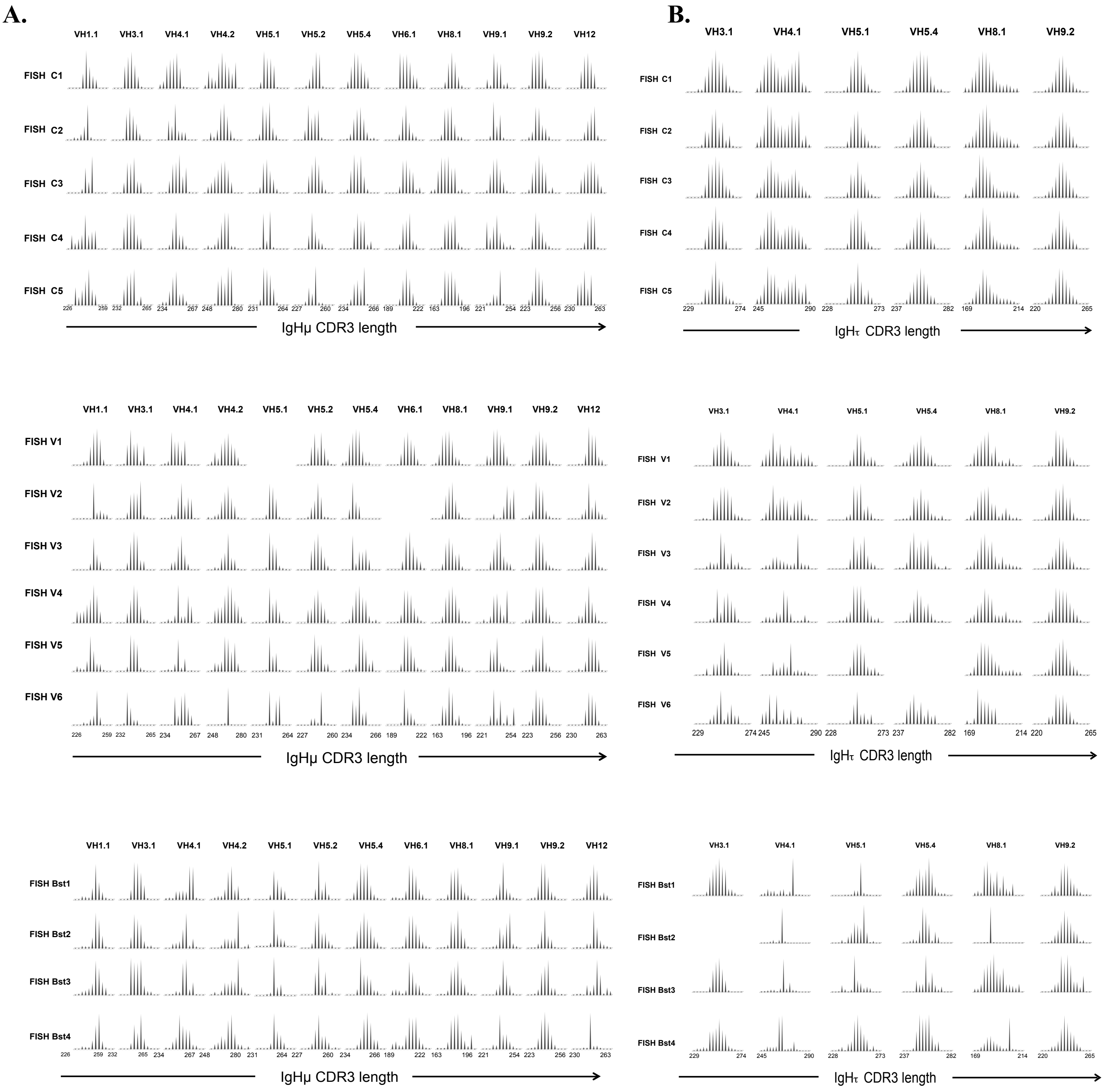

Supplement: Supplementary file 8 [file Image_3.pdf]
