# Supplementary material for: Origin of Public Memory B Cell Clones in Fish After Antiviral Vaccination
Source: Front Immunol. 2018 Sep 27;9:2115. doi: 10.3389/fimmu.2018.02115 (PMC6170628; doi:10.3389/fimmu.2018.02115)

Figure S4. Size distribution of clonotypes shared by individual sequenced repertoires from controls. (A) Size distribution is shown for each VH-C combination, and for clonotypes found in n control fish (n=1,2,3,4), within a subsampling of 7000 MID per fish. For clonotypes found in several fish, the average size is shown (B) Size distribution of TCLctl is represented in the same way. (C) Size distribution of VH5-Cμ clonotypes (and VH5-Cμ TCLctl clonotypes) found in n vaccinated fish (n=1,2,3,4) (D) Size distribution of VH5-Cμ clonotypes (and VH5-Cμ TCLctl clonotypes) found in n boosted fish (n=1,2,3,4). The eight public clonotypes involved in the response against VHSV are circled in black in panels C and D.

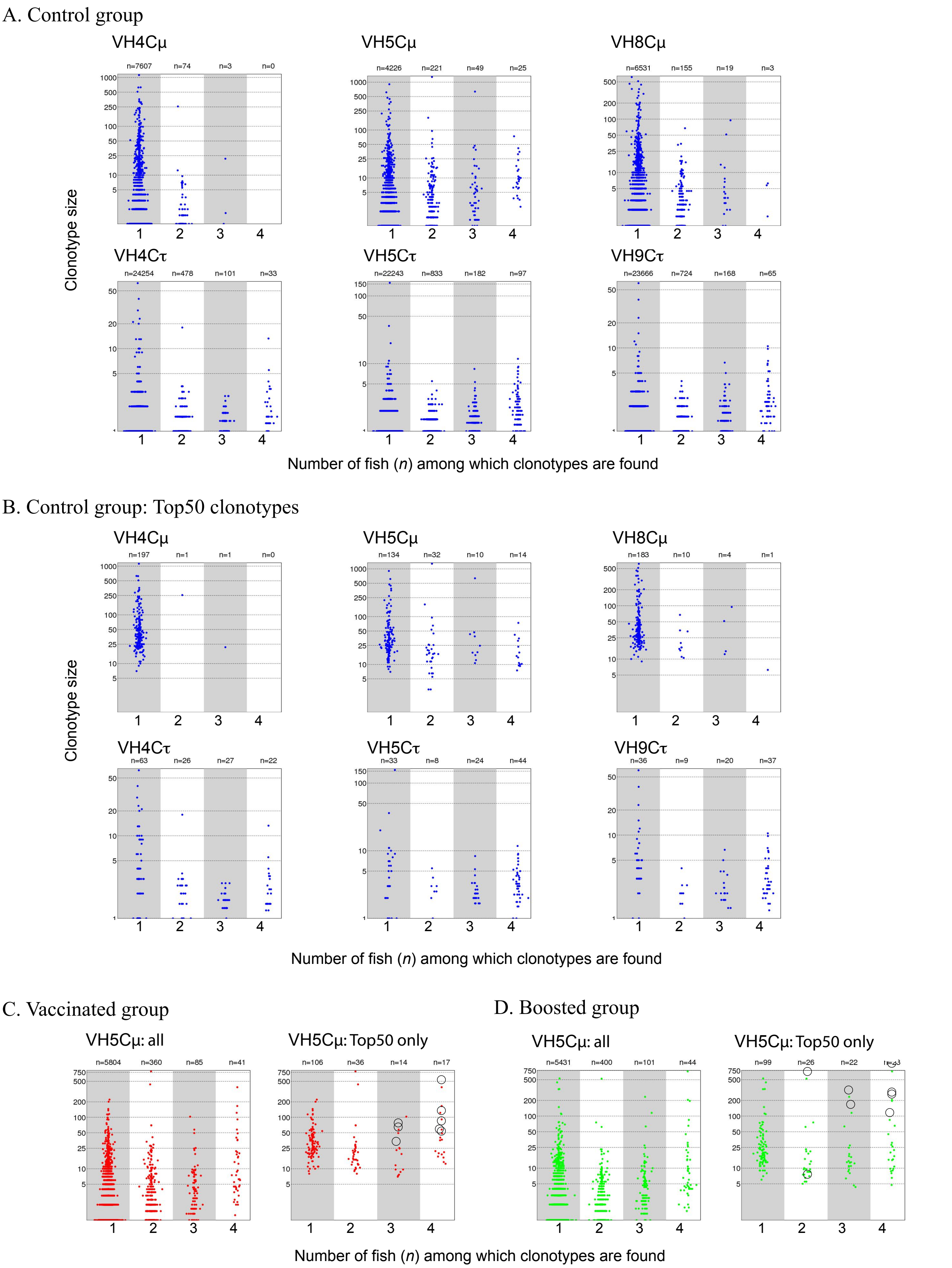

Supplement: Supplementary file 9 [file Image_4.pdf]
