# Supplementary material for: Origin of Public Memory B Cell Clones in Fish After Antiviral Vaccination
Source: Front Immunol. 2018 Sep 27;9:2115. doi: 10.3389/fimmu.2018.02115 (PMC6170628; doi:10.3389/fimmu.2018.02115)

Figure S5 A. Number of Top50 clonotypes shared by individuals within each group. (Ctl:control, Vac:vaccinated, and Bst:Boosted).

For each TCL (TCLCtl: a,d,g,j,m,p; TCLVac: b,e,h,k,n,q; TCLBst: c,f,i,l,o,r), bar plots showing the number of clonotypes found in individual subsample(s) from n fish (n=0,1,2,3,4), in the control group (blue bars), in the vaccinated group (red bars) and in the boosted group (green bars). For example, in panel a, blue bars show the numbers of TCLCtl clonotypes in fish from the control group, while red (respectively green) bars show numbers of TCLCtl clonotypes in fish from the vaccinated (respectively boosted) group. Similarly, in panel b, red bars show the numbers of TCLVac clonotypes in vaccinated fish, while blue (respectively green) bars show numbers of TCLVac clonotypes in control and boosted groups, respectively. Note that a Top clonotype from TCLCtl (panel a) can be absent from all fish from the Vac and Bst groups (see red and green bars in the column "0"). Bars are computed from the average values corresponding to top clonotypes found in 0 to 4 fish, over 10 subsamplings of 7000). The standard deviations are shown as error bars. On the right Venn diagrams showing the numbers of distinct Top50 clonotypes per fish group (TCLCtl, TCLVac, TCLBst), for each VHC combination.

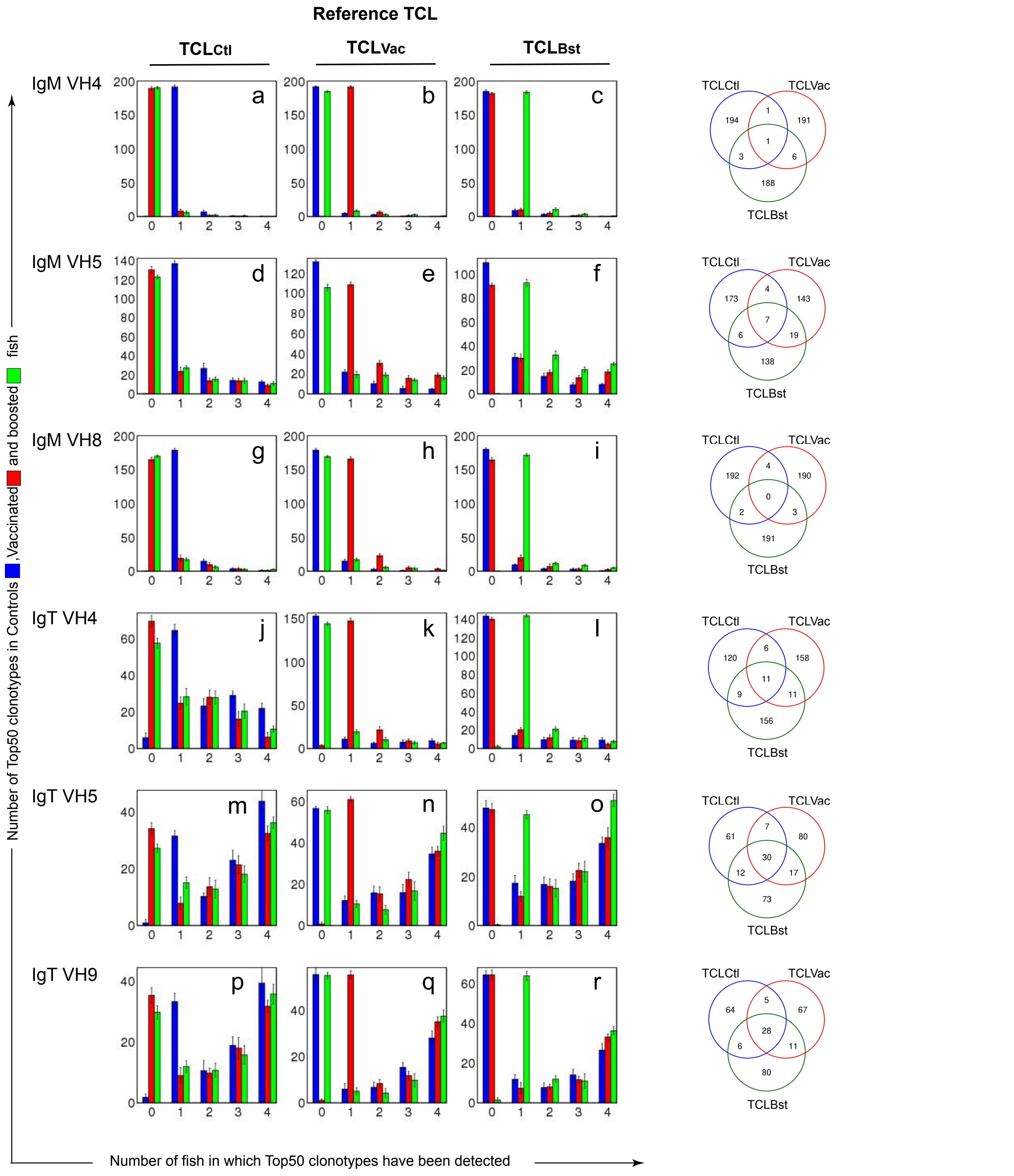

Figure S5 B. Number of Top100 clonotypes shared by individuals within each group. (Ctl:control, Vac:vaccinated, and Bst:Boosted). A. For each TCL (TCLCtl: a,d,g,j,m,p; TCLVac: b,e,h,k,n,q; TCLBst: c,f,i,l,o,r), bar plots showing the number of clonotypes found in individual subsample(s) from n fish (n=0,1,2,3,4), in the control group (blue bars), in the vaccinated group (red bars) and in the boosted group (green bars). For example, in panel a, blue bars show the numbers of TCLCtl clonotypes in fish from the control group, while red (respectively green) bars show numbers of TCLCtl clonotypes in fish from the vaccinated (respectively boosted) group. Similarly, in panel b, red bars show the numbers of TCLVac clonotypes in vaccinated fish, while blue (respectively green) bars show numbers of TCLVac clonotypes in control and boosted groups, respectively. Bars are computed from the average values corresponding to top clonotypes found in 0 to 4 fish, over 10 subsamplings of 7000). The standard deviations are shown as error bars.

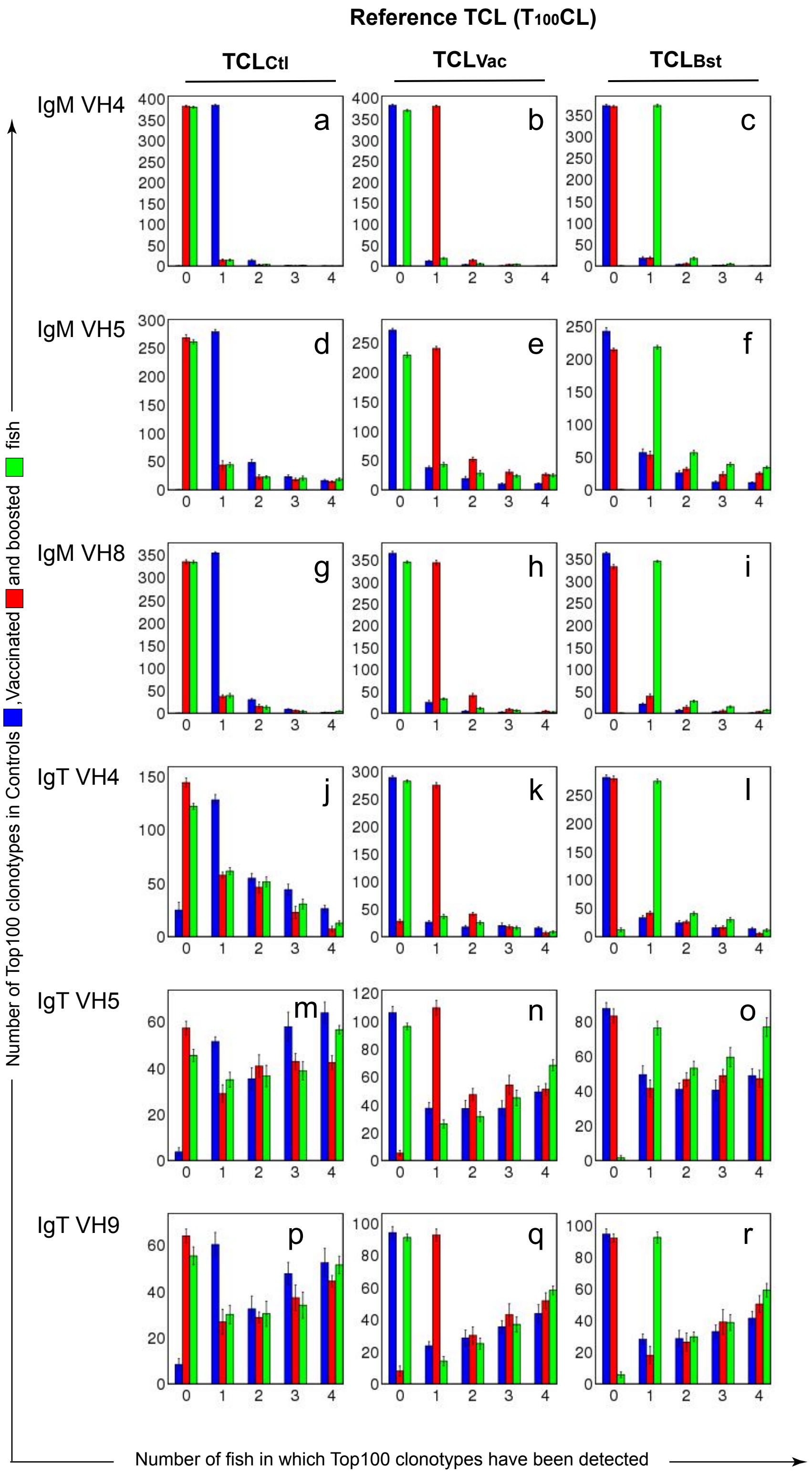

Figure S5 C. Number of clonotypes shared by n individual fish within each group (Ctl: control; Vac: vaccinated; Bst: boosted). Graphs are based on data from one subsampling; different subsamplings lead to similar results. The total number of distinct clonotypes present in the submaple is indicated in each panel.

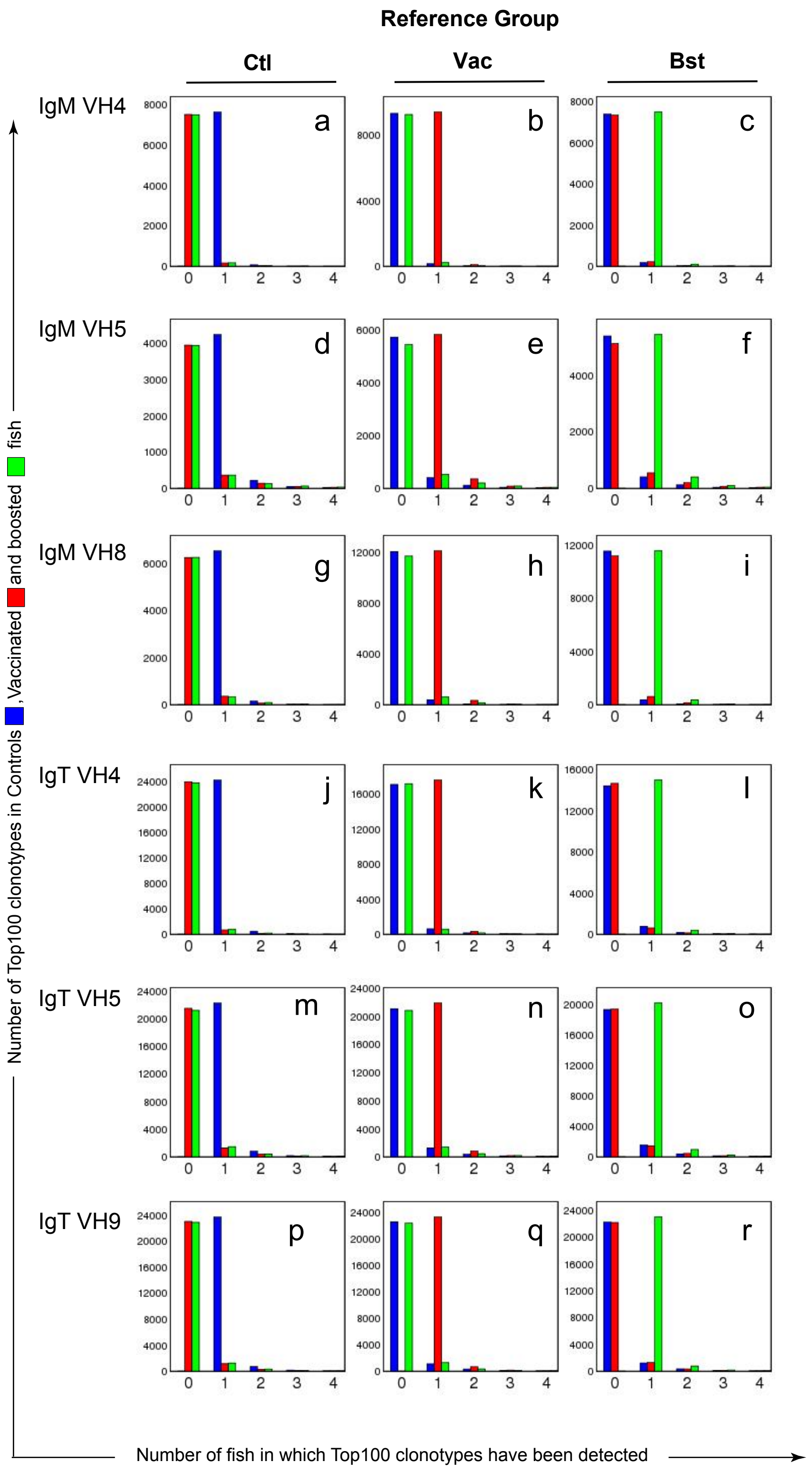

Supplement: Supplementary file 10 [file Image_5.pdf]
