# Supplementary material for: Origin of Public Memory B Cell Clones in Fish After Antiviral Vaccination
Source: Front Immunol. 2018 Sep 27;9:2115. doi: 10.3389/fimmu.2018.02115 (PMC6170628; doi:10.3389/fimmu.2018.02115)

Figure S6 A. Cumulative expression of Top100 clonotypes shared by n individuals within each group (Ctl:control, Vac:vaccinated, and Bst:Boosted) unveils differences between VH and isotypes. For each TCL (TCLCtl, panels a,d,g,j,m,p; TCLVac, panels b,e,h,k,n, q; TCLBst, panels c,f,i,l,o,r), bar plots showing the cumulative expression of TCL clonotypes found in individual subsample(s) from n fish (n=1,2,3,4). For example, in panel a, blue bars show the cumulative counts of TCLCtl clonotypes in fish from the control group, while red (respectively green) bars show TCLCtl cumulative expression in fish from the vaccinated (respectively boosted) group. Similarly, in panel b, red bars show the cumulative counts of TCLVac clonotypes in vaccinated fish, while blue (respectively green) bars show TCLVac cumulative expression in control and boosted groups, respectively. Bars are computed from the average values corresponding to top clonotypes found in 1 to 4 fish, over 10 subsamplings of 7000). The standard deviations are shown as error bars.

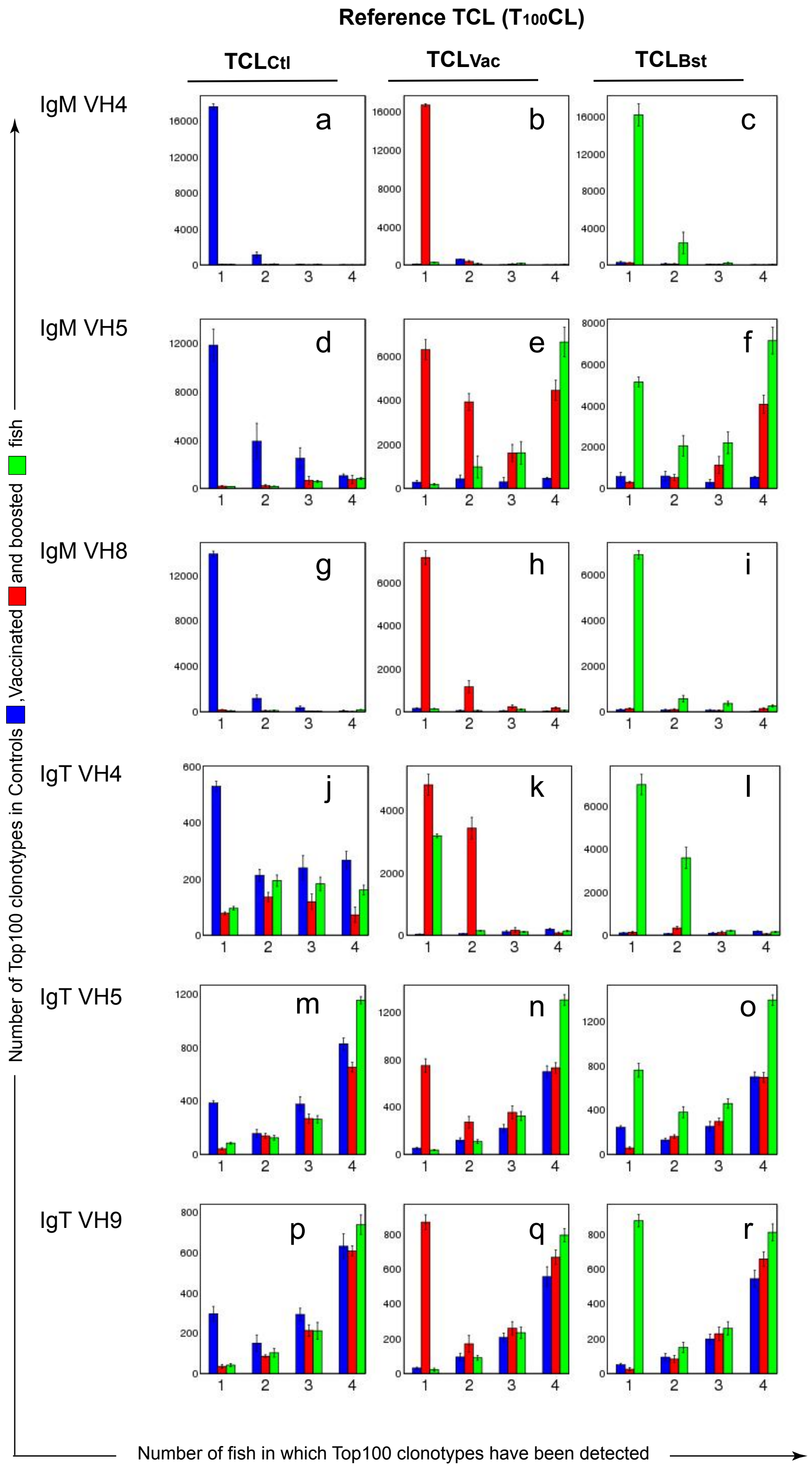

Figure S6 B. Cumulative expression of clonotypes shared by n individual fish within each group (Ctl: control; Vac: vaccinated; Bst: boosted). Graphs are based on data from one subsampling; different subsamplings lead to similar results.

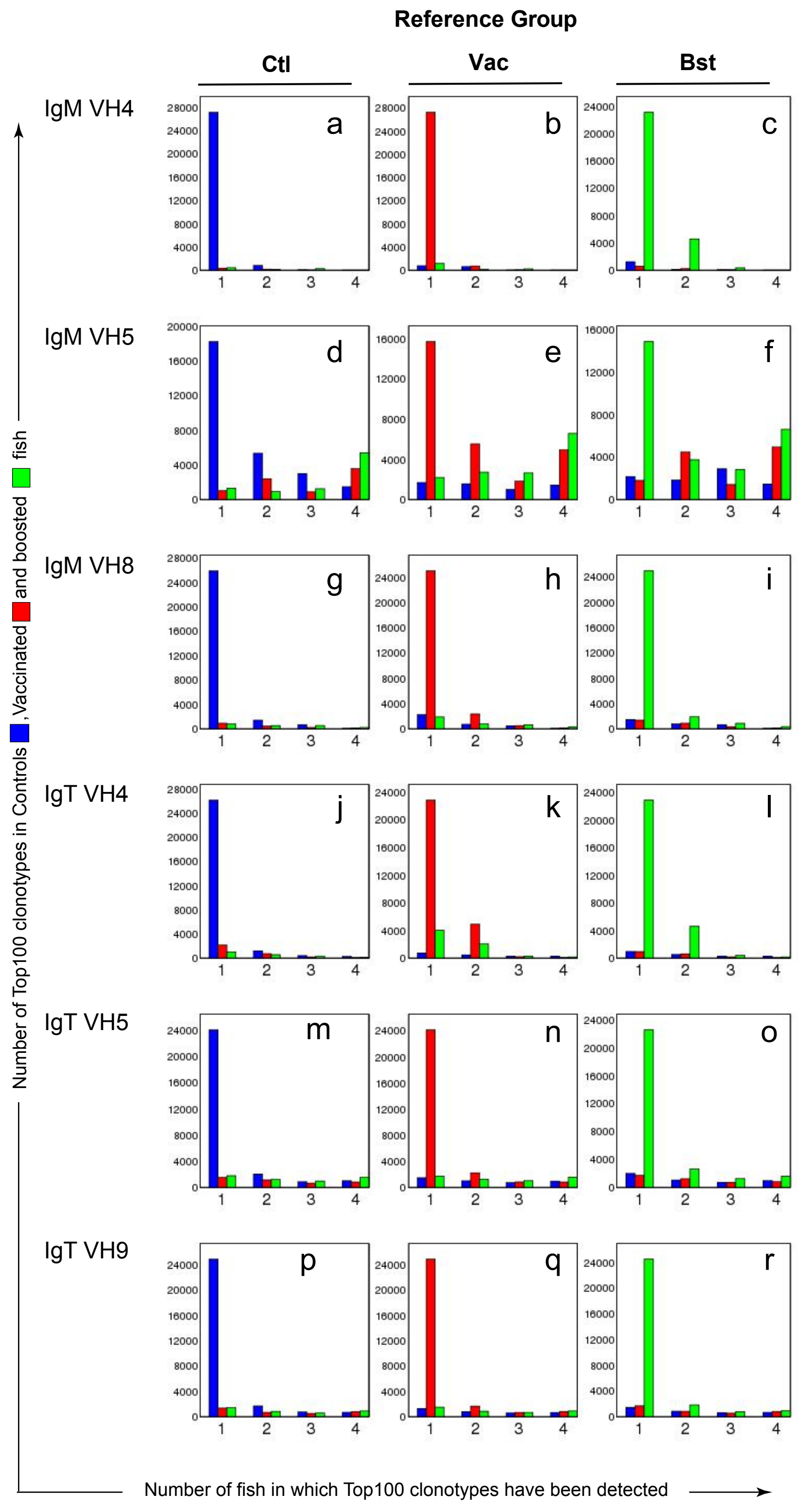

Supplement: Supplementary file 11 [file Image_6.pdf]
