# Supplementary material for: Origin of Public Memory B Cell Clones in Fish After Antiviral Vaccination
Source: Front Immunol. 2018 Sep 27;9:2115. doi: 10.3389/fimmu.2018.02115 (PMC6170628; doi:10.3389/fimmu.2018.02115)

Figure S7. Computational model of rainbow trout IgH VDJ rearrangements. Inferred distributions of rearrangements are shown.

A. Inferred D (left panel) and J (right panel) gene usage for each VH-C combination sequenced in this study (top row: IgM, bottom row: IgT). B. Distributions of insertion/deletion at the V-D (left panel) and D-J (right panel) joint, for each VHC combination. C. Distributions of generation probability (Pgen) for each VHC combination. The color code for VHC combinations is given on the right. The IMGT gene tables and nomenclature were used (ImMunoGeneTics database (www.imgt.org). Additional segments found in rainbow trout genomic data (BAC AY872256 and https://www.genoscope.cns.fr/trout/) were named by the accession number of the scaffold/BAC followed by the location of the gene segment. D. Histogramsof the sharing of all clonotypes (not only the TOP 50 as in Figure 4B) in subsamples and across VHC combination. Left (right) bars show the numbers of measured (synthetic) clonotypes shared among at least n individual measured (synthetic) repertoires (n =2,3,4) out of 4. The color code is given in the right and is consistent with the one used in Figure 4B.

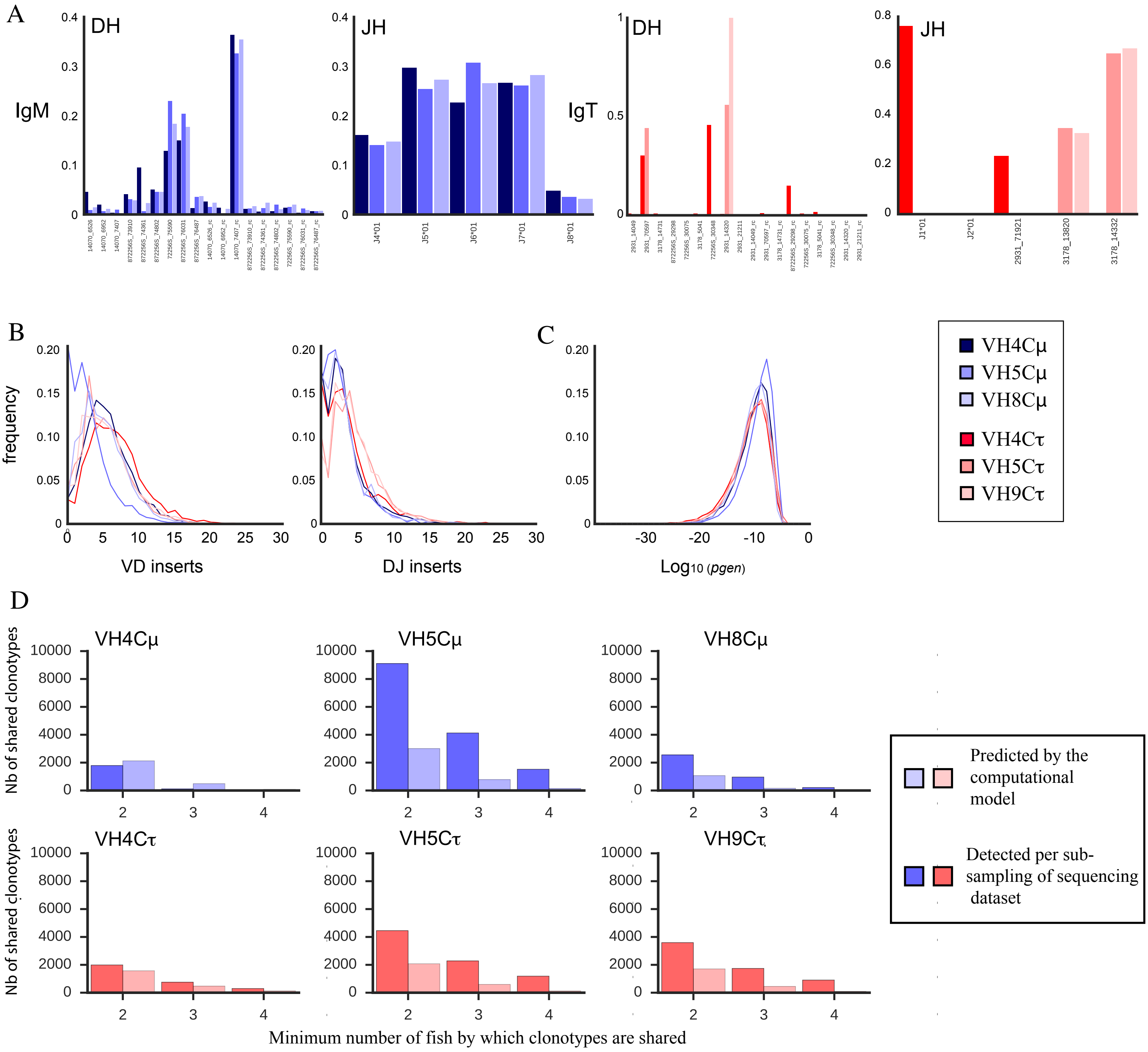

Supplement: Supplementary file 12 [file Image_7.pdf]
